# Realistic assumptions about spatial locations and clustering of premises matter for models of foot-and-mouth disease spread in the United States

**DOI:** 10.1371/journal.pcbi.1007641

**Published:** 2020-02-20

**Authors:** Stefan Sellman, Michael J. Tildesley, Christopher L. Burdett, Ryan S. Miller, Clayton Hallman, Colleen T. Webb, Uno Wennergren, Katie Portacci, Tom Lindström

**Affiliations:** 1 Department of Physics, Chemistry and Biology, Division of Theoretical Biology, Linköping University, Linköping, Sweden; 2 Zeeman Institute for Systems Biology and Infectious Disease Epidemiology Research, School of Life Sciences and Mathematics Institute, University of Warwick, Coventry, United Kingdom; 3 Department of Biology, Colorado State University, Fort Collins, Colorado, United States of America; 4 Center for Epidemiology and Animal Health, United States Department of Agriculture, Fort Collins, Colorado, United States of America; The University of Edinburgh, UNITED KINGDOM

## Abstract

Spatially explicit livestock disease models require demographic data for individual farms or premises. In the U.S., demographic data are only available aggregated at county or coarser scales, so disease models must rely on assumptions about how individual premises are distributed within counties. Here, we addressed the importance of realistic assumptions for this purpose. We compared modeling of foot and mouth disease (FMD) outbreaks using simple randomization of locations to premises configurations predicted by the Farm Location and Agricultural Production Simulator (FLAPS), which infers location based on features such as topography, land-cover, climate, and roads. We focused on three premises-level Susceptible-Exposed-Infectious-Removed models available from the literature, all using the same kernel approach but with different parameterizations and functional forms. By computing the basic reproductive number of the infection (*R*_*0*_) for both FLAPS and randomized configurations, we investigated how spatial locations and clustering of premises affects outbreak predictions. Further, we performed stochastic simulations to evaluate if identified differences were consistent for later stages of an outbreak. Using Ripley’s *K* to quantify clustering, we found that FLAPS configurations were substantially more clustered at the scales relevant for the implemented models, leading to a higher frequency of nearby premises compared to randomized configurations. As a result, *R*_*0*_ was typically higher in FLAPS configurations, and the simulation study corroborated the pattern for later stages of outbreaks. Further, both *R*_*0*_ and simulations exhibited substantial spatial heterogeneity in terms of differences between configurations. Thus, using realistic assumptions when de-aggregating locations based on available data can have a pronounced effect on epidemiological predictions, affecting if, where, and to what extent FMD may invade the population. We conclude that methods such as FLAPS should be preferred over randomization approaches.

## Author summary

When modeling the spread of infectious livestock diseases such as foot-and-mouth disease (FMD), the distance between premises is an important aspect. In the U.S., locations of premises are not available, forcing modelers to make assumptions about their coordinates. Such assumptions can be more or less crude and will impact the conclusions drawn from the model. To investigate the impact of such assumptions, we modeled outbreaks of FMD within the cattle population of the U.S. under two assumptions about premises locations. Their position was either randomly distributed within counties or informed by a state-of-the-art method developed specifically to simulate realistic locations of agricultural operations. We found that the higher degree of spatial clustering of premises associated with more realistic assumptions about locations leads to a substantially higher risk of outbreaks. Our results also show that the amount with which the risk is under-estimated by randomizing locations is unevenly distributed across the landscape. Together, these findings show a clear support for using informed methods to determine the spatial locations of premises and highlight the importance of spatial clustering when modeling FMD-like diseases.

## Introduction

Quantitative models for infectious livestock diseases are powerful tools for preparing and responding to disease incursions. Kermack and McKendrick’s Susceptible-Infectious-Removed (SIR) framework [1] and its extensions remain a foundation for disease modeling, but the increase in computational power over the recent decades has promoted the use of stochastic simulation modeling [2]. These simulation approaches can address specific policy questions and incorporate important deviations from the mass-action mixing assumption of the differential equation system of Kermack and McKendrick. For example, contacts that mediate transmission between premises are typically distance-dependent [3–5]; infections occur at a higher rate between nearby premises. Additionally, differences in transmission rates depending on premises types and sizes can have a pronounced effect on the course of an epidemic [6]. Consequently, the reliability of epidemiological models may depend heavily on the accuracy of information about premises distribution and demography.

Outbreaks of livestock diseases may be economically costly [7,8], impact animal and human health [9,10], and have severe consequences for affected communities [9,11]. Efficient control of emerging outbreaks can mitigate these consequences [12], and insights gained through modeling can be valuable. Through a mathematical description of the transmission process, modeling can aid disease preparedness and support policy decisions (e.g. by comparing control scenarios or identifying geographic hotspots of particular concern). Yet, the reliability of infectious disease models depends on their underlying assumptions and applicability to the focal system.

**Fig 1:**
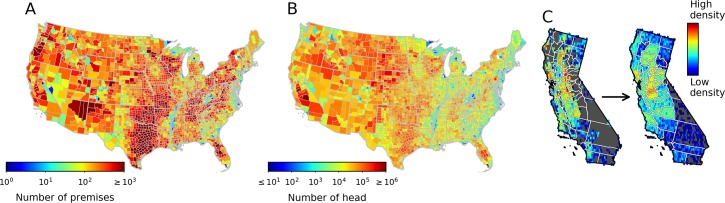
Spatial distribution of premises (A) and head (B) at the county level of the U.S. cattle population based on NASS [13,14] data common to all ten FLAPS realizations. Using California as an illustrative example, panel C shows how location randomization within counties (right) changes the spatial distribution of premises relative to the realistic assumptions used in FLAPS (left).

The United States (U.S.) livestock industry (Fig 1A and 1B) is dominated by cattle, with more than 900,000 premises and approximately 103 million animals [13,14]. In 2015, the industry accounted for USD$78.2 billion in cash receipts [15]. An outbreak of a transboundary animal disease such as foot and mouth disease (FMD) would have severe economic impacts on the industry, particularly due to disruption in trade [7]. Stakes are high for policy decisions, yet modeling efforts to promote disease preparedness are challenged by limited demography data. Premises-level data describing location, animal inventory, and premises type is not uniformly collected for the U.S. livestock industries due to stakeholder concerns regarding implementation cost as well as confidentiality and security of collected information [16,17]. A voluntary system maintained by the U.S. Department of Agriculture currently contains approximately 25% of the estimated 1.4 million livestock and poultry premises in the U.S. [18].

The most comprehensive inventory of U.S. livestock demography is provided by the National Agriculture Statistics Service (NASS), which conducts surveys of the livestock and poultry industries and provides demographic data describing animal inventory and number of premises aggregated at the county level. Thus, there is an absence of detailed information about the spatial distribution of the premises, prompting livestock disease modelers to assume either randomly distributed premises [19] or mass-action mixing [20] within counties. However, there may be problems associated with such assumptions. Based on spatial patterns found in other countries, premises are expected to be clustered at spatial scales finer than the sizes of the counties [5,21].

As such, conclusions drawn from crude approximations about premises locations may be misleading. When premises are clustered, there is a higher frequency of short distances between premises compared to when locations are random. Disregarding such clustering may under-predict transmission when distance-dependent transmission rates are assumed. On the other hand, clustered premises distributions could cause the disease to remain within a cluster, effectively trapping the infection process and leading to more contained outbreaks. Modeling studies in the U.K. suggest that using land-cover maps to impute locations improves the predictive accuracy of epidemiological models [22], but randomized premises locations may be sufficient for identifying optimal control [21]. However, compared to the U.K., the U.S. is more geographically heterogeneous, and the large spatial scale allows for more heterogeneous clustering patterns across the continent.

Recent years have seen a rise in computational methods that predict missing or withheld information about the U.S. livestock industry based on the information that is available. One such method is the Farm Location and Agricultural Production Simulator (FLAPS) [23], which uses the county-level premises size distributions provided by NASS in combination with environmental, geographical, and anthropogenic predictors (e.g. climate, topography, land-cover, and roads) to predict explicit premises locations. While not a true depiction of U.S. livestock premises, FLAPS offers the most detailed predictor of the premises distribution across the country, and validation efforts have shown a high correlation between predicted and existing premises locations [23].

In this study, we aimed to answer a simple yet crucial question for spatially explicit livestock modeling in countries where information on demography is limited: do realistic predictions about premises locations matter? To answer this, we used spatial distributions of premises as predicted by FLAPS, and landscapes where the premises’ locations were randomized within their respective counties, together with three implementations of previously published FMD models. The models all shared the same premises-level, kernel-based structure that was first developed for the 2001 FMD outbreak in the UK [4] and are referred to by the first name of the original publications, Brand [19], Hayama [3] and Tildesley [24]. Using these models, we performed two different analyses to evaluate the importance that detailed premises locations would have for the dynamics of a potential FMD outbreak within the U.S cattle population. The first was an analytical approach where we calculated the basic reproductive number, *R*_*0*_, for disease transmission in the two landscape configurations, allowing us to compare the risk of an FMD outbreak across the U.S. under different assumptions about spatial clustering and analyze the effect of within-county clustering on risk dynamics. The second approach was to perform stochastic simulations of FMD outbreaks which enabled us to investigate the effect of realistic spatial clustering for the later stages of outbreaks.

## Results

### Characterization of the spatial distributions of premises and quantification of clustering

**Fig 2:**
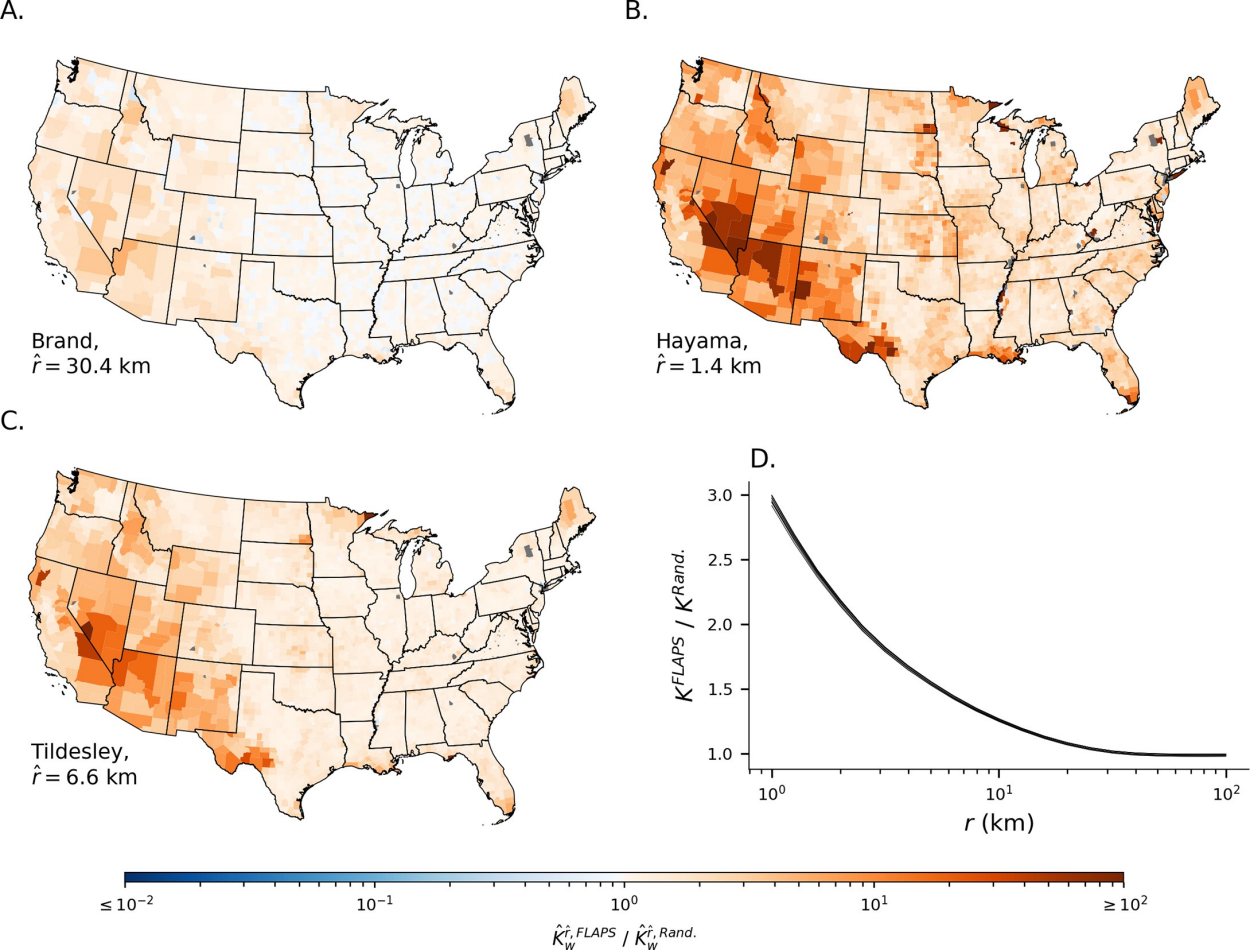
Landscape-level clustering. (A-C) Relative change in county level clustering, ${\hat{K}}_{w}$, when going from randomized configuration to FLAPS for spatial scale $\hat{r}$ where the respective kernel functions had fallen by 95% of *H(0)*. (D) Landscape-level relative change in clustering measured as Ripley’s *K* at different radii when going from randomized configuration to FLAPS. The panel shows ten nearly indistinguishable lines, each indicating the difference between one FLAPS realizations and its randomized counterpart.

**Fig 3:**
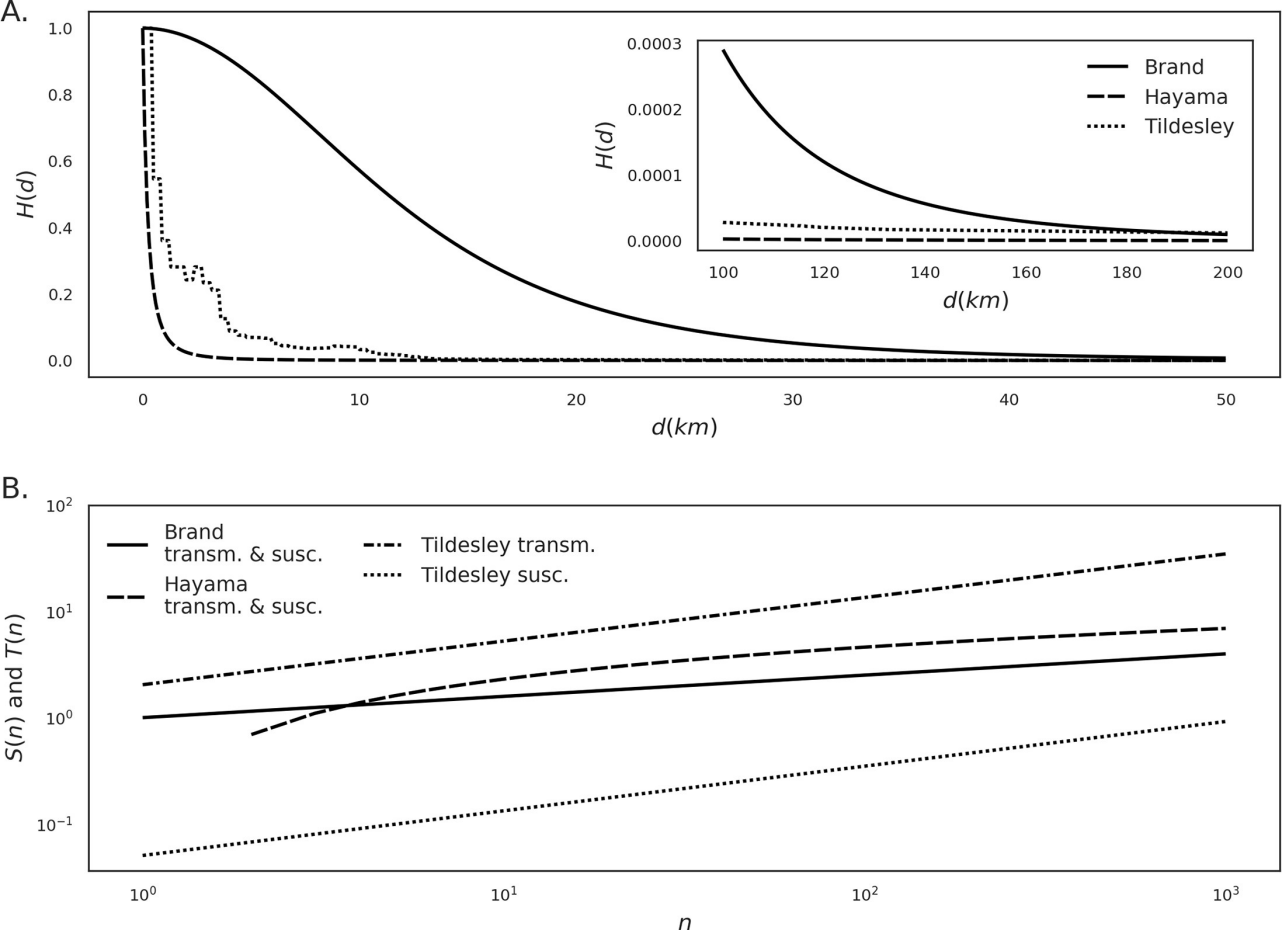
Functional forms of the distance kernel *H* as a function of the distance in kilometers (A), and transmissibility (*T*) and susceptibility (*S*) as functions of herd size (*n*, panel B). To enable comparison, the kernels have been scaled (for the purpose of this plot only) so that *H(0) = 1*.*0*.

In the U.S., reliable, national-scale data on the spatial distribution of cattle and premises with cattle are not available at a finer scale than the individual county (Fig 1A and 1B). Thus, ten simulated distributions were generated with FLAPS [23], representing populations of premises with realistic degree of spatial clustering. For each of these realizations, a matching distribution was generated by randomizing each premises’ location within its county (Fig 1C). We refer to these sets as FLAPS and random configurations (available as S1 Dataset and S2 Dataset). Spatial clustering within these populations was quantified at the landscape level for different spatial scales *r* using Ripley’s K [25], denoted *K*^*r*^. We also used a variation of Ripley’s K, denoted $K_{w}^{r}$, that promotes quantification of average clustering of premises in each county *w* for spatial scales *r*, and we let ${\hat{K}}_{w}^{r}$ denote the arithmetic average of $K_{w}^{r}$ across the ten realizations of each of the two configurations. Overall, spatial clustering was higher in FLAPS configurations compared to randomized configurations (Fig 2). The difference was most pronounced at shorter distance scales, with the relative difference in clustering, *K*^*r*,*FLAPS*^/*K*^*r*,*Rand*.^, three times higher at 1 km and dropping off to imperceptible differences (between 0.989 and 1.007 relative difference depending on realization) at 40 km (Fig 2D). To evaluate clustering differences at spatial scales at which we would expect FMD transmissions to be likely, we defined $\hat{r}$ for each of the three FMD models as the distance where the transmission kernel had dropped to 5% of its value at distance *d = 0*. The shapes and functional forms of the transmission kernels are two of the major differences between the models that were used and controls how the risk of transmission relates to the distance between premises (Fig 3A). Consequently, the largest relative difference in clustering was observed at the scale most relevant to the Hayama kernel ($\hat{r}$ = 1.4 km), followed by the scale of Tildesley ($\hat{r}$ = 6.6 km) and Brand ($\hat{r}$ = 30.4 km). Fig 2A–2C also reveals substantial geographical heterogeneity regarding how the realistic assumptions about premises locations included in FLAPS affects spatial clustering at the county level. In large counties, typically in the western U.S., clustering at the scales relevant for the three implemented models was substantially higher in the FLAPS configurations than the randomized. For the Hayama scale, there were also substantial differences in small counties in the central states, a pattern found also for the Tildesley scale, however with less intensity.

### Basic reproductive number, *R*_*0*_

**Fig 4:**
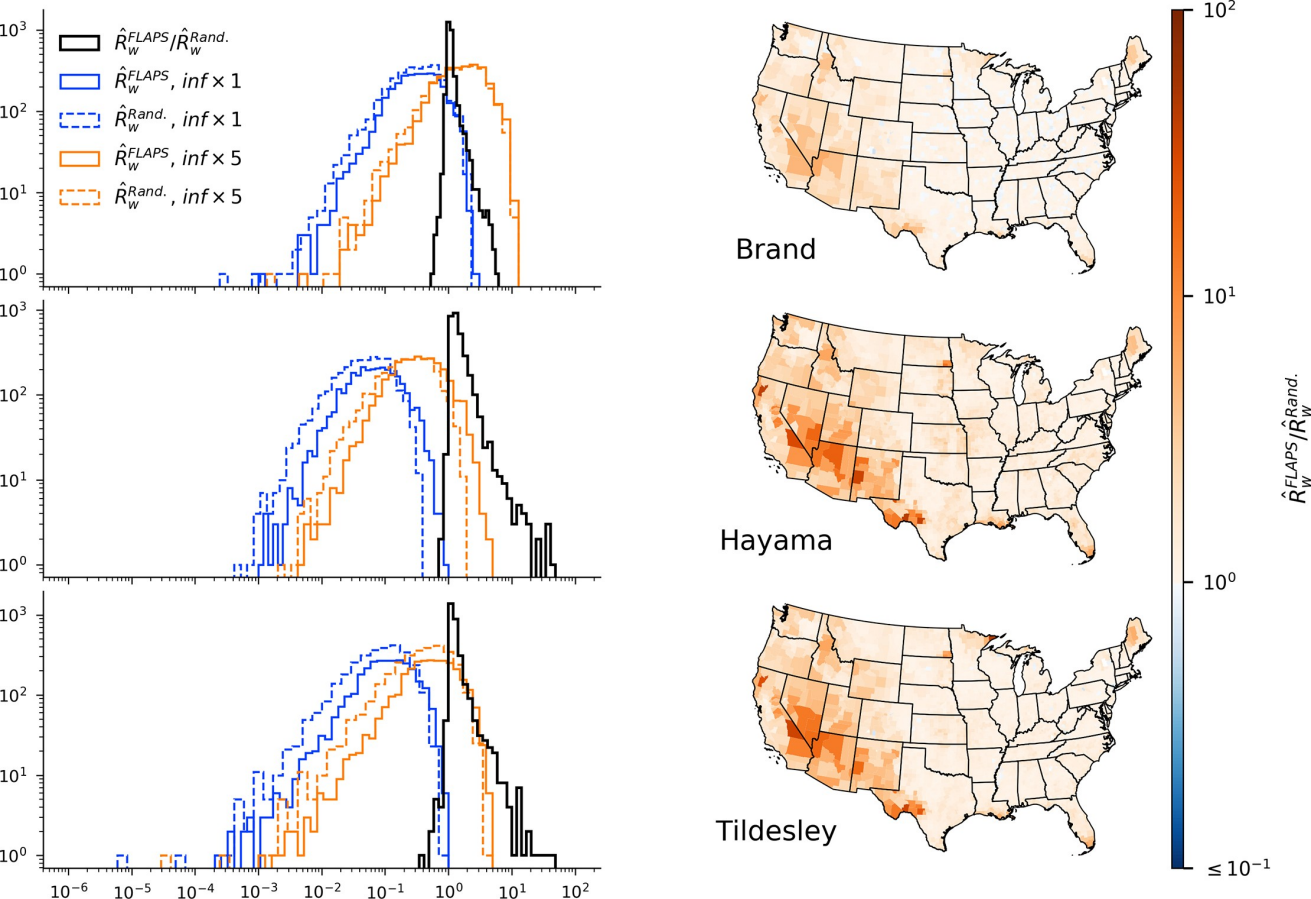
Frequency and spatial distributions of ${\hat{R}}_{w}$ and relative difference in ${\hat{R}}_{w}$ between FLAPS and random configurations. Geometric mean *R*_*i*_ across the premises of each county *w* for FLAPS (${\hat{R}}_{w}^{FLAPS}$) and randomized (${\hat{R}}_{w}^{Rand.}$, dashed) and configurations (left). Frequency distribution of the proportional difference in ${\hat{R}}_{w}$ between configurations shown by the black line in histograms (left) and its spatial distribution is illustrated by the maps. The proportional difference shown by the black histogram is independent of the increase applied to the transmission rate.

The basic reproductive number, *R*_*0*_ is the expected number of secondary cases following infection of a given premises *i* (*R*_*i*_) and is derived analytically based on the assumptions of a given model. Using the geometric mean of *R*_*i*_ across the premises of county *w* in the ten realizations of each of the configurations, we evaluated the effect of clustering on the risk that an introduction of the pathogen into *w* leads to an actual outbreak. With the original parameterizations of the models we found that for most counties ${\hat{R}}_{w}$ was low (Fig 4), indicating that an outbreak is generally not expected, should FMD be introduced at a random premises. To provide an illustration of an alternative parameterization with a higher risk we also performed the same calculations with the models’ infection rates increased by a factor of five. Regardless of parameterization, the *R*_*0*_ estimates showed substantial variation, both among premises within configuration sets and between configuration sets (see figures S1 Fig and S2 Fig for analyses using minimum and maximum *R*_*0*_ per county instead of the geometric mean). Clustering had a marked effect on the outbreak risk; ${\hat{R}}_{w}$ based on FLAPS configurations were higher than randomized for most counties with 80.3%, 99.3% and 98.0% increase for the Brand, Hayama and Tildesley models respectively. For the same models, 2.8%, 15.0% and 9.2% of the counties’ ${\hat{R}}_{w}$ increased to twice or more in the FLAPS configurations.

Importantly, using FLAPS as opposed to randomized configurations increased the proportion of counties with an ${\hat{R}}_{w}$ above one, i.e. where an outbreak is expected if the pathogen is introduced. The effect varied among models and parameterizations, ranging from a proportional increase of 5.6% for the Brand kernel with transmission rate × 5, to 191.2% for the Hayama kernel with transmission rate × 5. For the parameterization without increase to transmission rate both the Hayama and Tildesley kernels lacked any counties with ${\hat{R}}_{w}$ over one, regardless of landscape configuration. For the Brand kernel, the increase due to landscape configuration using the original transmission rate was higher (20.2%) than for the parameterization using the increased transmissibility.

The effect of realistic assumptions about spatial clustering modeled with FLAPS varied among U.S. counties, and across all models. There was a substantial fraction of counties where the FLAPS configurations resulted in an ${\hat{R}}_{w}$ of more than an order of magnitude than their randomized counterpart. These differences between configurations were positively correlated with the area of the county (S3 Fig) and followed the same geographical pattern as differences in terms of clustering. However, in particular with the Hayama model, there was also a substantial increase in ${\hat{R}}_{w}$ in small, centrally located counties.

In the supplement we show that there was little variation across the ten different FLAPS-realizations (S4 Fig).

### Outbreak simulations

Stochastic simulations with each of the three models were performed by seeding infection at one premises 1,000 times in each county. The seeded premises was selected at random for each replicate, and the outbreak was simulated until 100 premises were infected, or the outbreak died out. We chose to end simulations at this cut-off in order to reduce computational times and allow a larger amount of replicates. In the supplemental material we show that based on seedings in a subset of counties (one per state), most simulated outbreaks that reached 100 infected premises also reached much larger number of infected premises (>10,000), indicating that the cut-off of 100 premises is an appropriate proxy for large outbreaks (S5 Fig).

**Fig 5:**
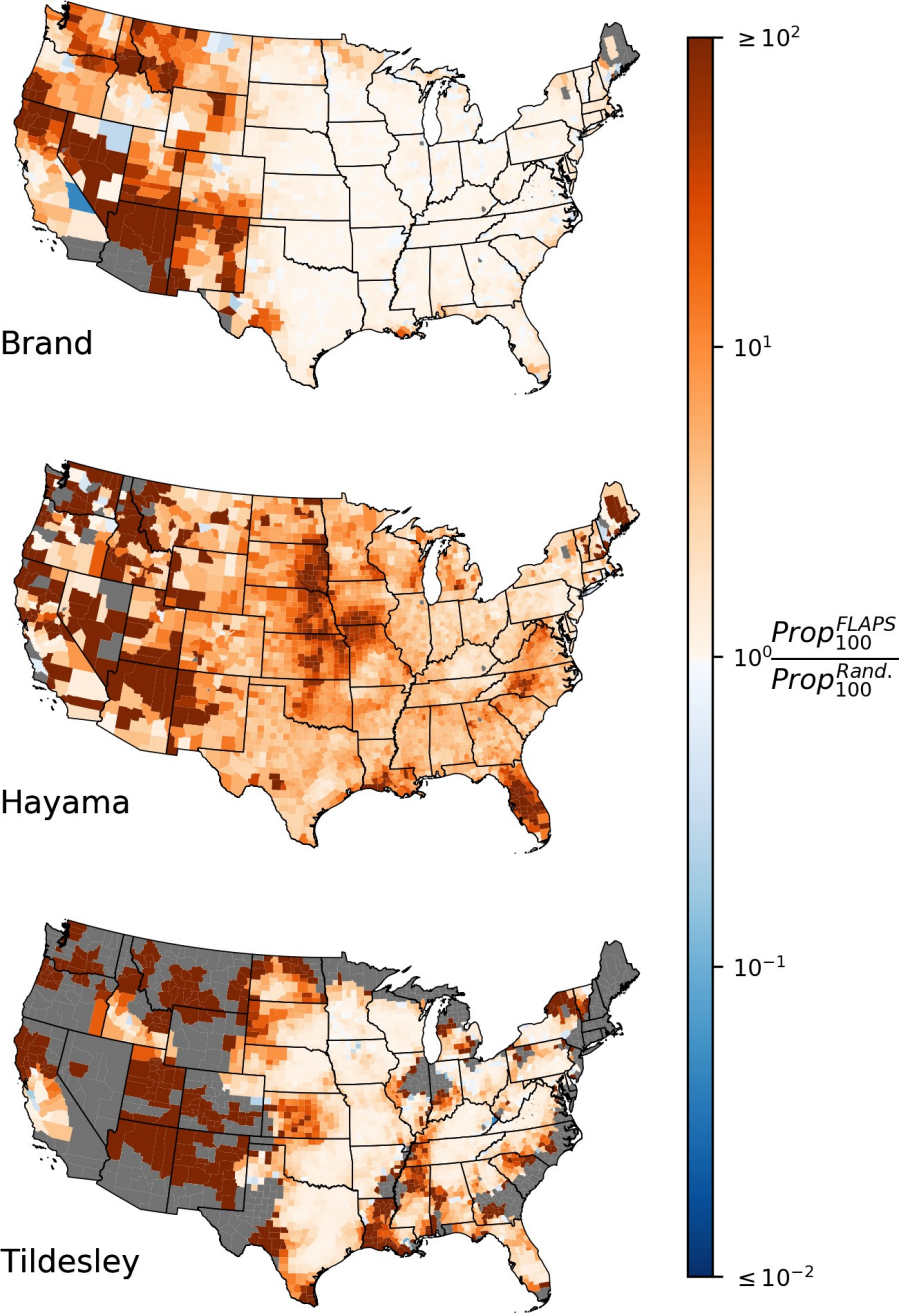
Differences in probability of large outbreaks. **Transmissibility x5.** County-level proportional change in number of replicates that reached 100 infected premises when using FLAPS compared to randomized configurations. Grey indicates counties where no replicate reached 100 infected in either FLAPS simulations or randomized simulations or both. Results are based on kernel parameterizations with five-fold increase in transmissibility.

Because ${\hat{R}}_{w}$ was typically below one for most counties when using the original parameterizations of the models, only a minority of the replicates generated secondary infections. This posed a problem for analyzes of later stages of the outbreaks, and we therefore focus on simulations where the transmission rate was amplified by a factor five to investigate if differences in ${\hat{R}}_{w}$ translated to differences in outbreak simulations. Based on these simulations, we found that the frequency of large outbreaks (>100 premises) was substantially higher in the FLAPS-generated configurations compared to the random configurations (Table 1; S1 Table for the subset of simulations ≥10,000 infected premises, S1 Appendix for an analysis of the maximal outbreak size). The general spatial pattern of the increase in outbreaks reaching 100 infected premises when using FLAPS over random landscapes matched that of the corresponding increase in county-level clustering (Fig 5).

A linear regression showed that the probability of reaching at least two infected premises (including the index case) in the simulations was strongly related to the ${\hat{R}}_{w}$ of the seeded county, as shown by an *R*^2^ value of between 0.93 and 0.95 for the different kernels (S6 Fig). The explanatory power of ${\hat{R}}_{w}$ of the seeded county to predict outbreaks reaching at least ten infected premises was somewhat lower in comparison (*R*^*2*^ between 0.65 and 0.78) and for 100 infected premises fell to between 0.34 and 0.56.

To test the effect of clustering and configuration on the risk of obtaining large outbreaks, we performed a logistic regression for each of the three kernels with the Bernoulli trial of reaching 100 cumulative infected premises or not as the dependent variable. Out of the predictors in the logistic regression, we found that clustering of the county with the seeded premises was the factor most important to explain the probability of a large outbreak. The logistic regression showed that for all three kernels, removing clustering as a predictor variable resulted in the largest decrease in explanatory power (Tjur’s coefficient of discrimination, see “Simulations” in the methods section) by far compared to the full model (Table 2). The detailed results from the logistic regression can be found among the supplementary materials (S2 and S3 Tables). In the supplementary material we show that the number of time steps needed to reach 100 infected premises was similarly affected by clustering and landscape configuration (S4 Table). Figures summarizing the results from the outbreak simulations and the analyses without amplified transmissibility are presented in the supplementary material (S7–S11 Figs).

**Table 1:** The proportion of simulated outbreaks that reached 100 infected premises. Each combination of kernel, transmissibility scale factor and spatial premises distribution is based on 30,490,000 seeded outbreaks.

	Random		FLAPS	
Kernel	Transm. x 1	Transm. x 5	Transm.x 1	Transm.x 5
Tildesley	0.00003	0.14809	0.00252	0.21220
Hayama	0.00000	0.02483	0.00041	0.09530
Brand	0.04176	0.54205	0.05377	0.57805

**Table 2:** Coefficient of discrimination (*CoD*) and relative change in *CoD* (*ΔCoD)* as predictors are removed from logistic regression models predicting the probability of reaching 100 infected premises. The predictors are the county-level clustering at a spatial scale relevant for the kernel, $K_{w}^{\hat{r}}$ *(Clustering)*, the type of configuration (random or FLAPS, *Landsc*. *conf*.), the logarithm of the average premises size in the seeded county, $ln\;{\bar{n}}_{w}$ *(Avg*. *prem*. *size)*, the logarithm of the number of premises in the seeded county (*N*. *prem*.), *ln N*_*w*_ and the logarithm of the premises size of the seeded premises, *lnn*_*i*_ (*Seeded size)*. The model coefficients for these predictors as well as nine additional binary predictors indicating which out of ten different realizations of the landscape configuration are found in the supplementary material (S2 Table and S3 Table).

		*Transmissibility x1*		*Transmissibility x5*	
	*Removed variable*	*CoD*	*ΔCoD*	*CoD*	*ΔCoD*
Brand	*None (full model)*	0.373	0.000	0.533	0.000
	*Clustering*	0.153	0.590	0.274	0.486
	*Landsc*. *conf*.	0.370	0.008	0.532	0.001
	*Avg*. *prem*. *size*	0.370	0.008	0.526	0.012
	*N*. *prem*	0.372	0.003	0.532	0.002
	*Seeded size*	0.365	0.022	0.519	0.026
Hayama	*None (full model)*	0.117	0.000		0.329	0.000
	*Clustering*	0.014	0.876	0.174	0.470
	*Landsc*. *conf*.	0.119	-0.020	0.329	-0.001
	*Avg*. *prem*. *size*	0.115	0.014	0.329	-0.001
	*N*. *prem*	0.113	0.033	0.327	0.006
	*Seeded size*	0.112	0.041	0.318	0.034
Tildesley	*None (full model)*	0.128	0.000	0.438	0.000
	*Clustering*	0.027	0.790	0.263	0.399
	*Landsc*. *conf*.	0.129	-0.009	0.437	0.001
	*Avg*. *prem*. *size*	0.112	0.131	0.405	0.075
	*N*. *prem*	0.128	0.003	0.438	0.000
	*Seeded size*	0.116	0.100	0.400	0.086

## Discussion

Applied epidemiological modeling is challenged by limited data, either because information is withheld due to confidentiality concerns or because the data has not been collected. In the absence of exact information regarding premises demography and contact patterns, modelers have to extrapolate from available information and make assumptions about spatial distributions [19,22,26]. Here we considered potential for FMD transmission within the U.S. cattle industry, where publicly available demography data are aggregated to the county level. Small-scale spatial clustering can have a pronounced effect on epidemiological invasions [5,27], suggesting that realistic assumptions about farm locations within counties could have important implications for epidemiological predictions. This has, however, never been comprehensively addressed.

The absence of large-scale outbreaks in the U.S. prevents us from addressing predictive accuracy in terms of the ability to reproduce observed dynamics. We may however investigate the added value of techniques that estimate detailed premises locations by comparing simulated epidemics on such premises populations to simulations using cruder estimates of premises distributions. Here, we focused on premises configurations generated by FLAPS, which predicts spatially explicit premises locations and premises sizes based on survey data, environmental variables and anthropogenic factors, and compared *R*_*0*_ and simulated outbreaks to configurations where locations were randomized within counties. At present, FLAPS provides the most detailed and, arguably, realistic depiction available of the entire U.S. cattle farm and feedlot population.

At scales relevant for the implemented models, spatial clustering was substantially higher in FLAPS configurations compared to randomized counterparts. For instance, the *K*^*FLAPS*^*/K*^*Rand*^ ratio of around three found at 1km in Fig 2D indicates that premises have on average three times as many farms within a 1km radius in FLAPS configurations. However, differences between configurations exhibited large variation among counties, with some counties exhibiting differences between configuration types of more than two orders of magnitude at the scales relevant for the Hayama and Tildesley kernels (Fig 2A–2C).

The observed differences in spatial clustering between configurations translated into differences in terms of disease transmission. Across the three implemented models, county-level reproductive number, ${\hat{R}}_{w}$ was generally larger for FLAPS configurations compared to their randomized counterparts (Fig 4). The geographic pattern of differences in ${\hat{R}}_{w}$ corroborated with differences in local clustering, and ${\hat{R}}_{w}$ was considerably higher for FLAPS configurations for many counties, in particular for the Hayama and Tildesley kernels. Also, calculations based on FLAPS configurations had a pronounced effect on the proportion of counties with ${\hat{R}}_{w}$ >1. This suggests that using realistic assumptions about spatial locations are important to evaluate if and where FMD outbreaks are expected following introduction.

However, ${\hat{R}}_{w}$ does not directly estimate the transmission risk beyond the initial stage of an outbreak, and we used simulations to explore the risk of large outbreaks, here defined as at least 100 infected premises. A priori expectations regarding the effect of increased clustering for the progression of an outbreak are not straightforward. Two conflicting processes could be proposed: 1) the disease may get stuck in a local cluster, thus decreasing the risk of large outbreaks, or 2) the higher frequency of nearby farms promotes higher rates of transmission also for later stages of the outbreak. The patterns illustrated by Fig 5 shows that the latter process prevailed over the former in our simulations; only for a handful of counties was the risk of an outbreak reaching 100 infected premises higher for randomized configurations. This general increase in the risk of outbreaks reaching 100 infected premises, together with the support for such outbreaks to commonly grow even larger (S5 Fig), clearly shows that the increased clustering imposed by realistic assumptions regarding spatial locations increases the risk of large outbreaks.

The results of our analyses were based on a range of published FMD kernel models. The Tildesley kernel is derived from the U.K. 2001 outbreak and has been modified to fit with U.S. conditions of a single state (Pennsylvania) [24]. The Hayama kernel, which was originally fitted to a Japanese outbreak, was used without modification beyond the implementation of a fixed infectious period, which was necessary for the *R*_*0*_ calculations. The Hayama kernel represents a case of steep decay of transmission rate with distance, which may be unrealistic for U.S. conditions, where premises are spatially separated by larger distances. Conversely, the Brand kernel corresponds to an assumption of disease transmission over large spatial scales. As there has not been an FMD outbreak in the U.S. in recent years, there is no outbreak data to parameterize models from. Consequently, parameter values for any U.S. FMD model are currently well-educated guesses at best. Thus, neither of the models implemented here are meant to provide meticulous expectations with respect to the risk or extent of FMD outbreaks in the U.S. and for this purpose, the results should be interpreted with caution. However, our aim was not to make predictions about FMD outbreaks in the U.S. but to analyze the effect of spatial clustering for such predictions. Because our results are based on a range of kernels that differ in terms of transmission rates (including the increase by a factor 5), distance dependence, effect of premises size, and infective period, the conclusions of our study are robust to a wide range of assumptions for FMD-like diseases in the U.S.

Several patterns were consistent across models, with realistic spatial locations, as predicted by FLAPS, generally increasing clustering at scales relevant for all three models. These differences translated into increased *R*_*0*_ and higher frequency of large outbreaks and rapid spread, especially in large, western counties. Dispersed randomly, distances between premises are often too large to promote disease spread, but when clustered to regions that permit livestock production, premises locations allow for frequent transmission. The Brand kernel implements the slowest decay of transmission rate with distance (Fig 3), and it is not surprising that the effect of FLAPS on clustering—and subsequently *R*_*0*_ and simulated outbreak risk—was the lowest for this kernel (Fig 2; Fig 4, top panels). With increased spatial coverage of the kernel, the effect of local spatial configuration decreases. However, the general pattern, with a substantial effect of realistic spatial configuration is robust across the implemented kernels.

Our analysis is the first to consider the epidemiological implications of realistic spatial clustering of premises for the entire U.S. It corroborates theoretical findings from previous studies that spatial clustering of premises is essential for estimating epidemics [5,27]. Across models, the logistic regression analysis (Table 2, S2 & S3 Tables) identified clustering as an essential factor for the probability of large outbreaks. It should be noted that although the regression shows no particular effect of the variable *landscape configuration*, this does not mean the landscape configuration has no effect on the probability of reaching 100 infected premises. That effect is captured in the variable $K_{w}^{\hat{r}}$ (*clustering*), which is the explicit county-level clustering, and consequently *landscape configuration* only captures differences between the configuration types driven by other factors than county-level clustering. Instead, the regression analysis pinpoints the important of accurate depiction of spatial clustering of premises.

Tildesley et al. [21] showed that randomization of premises locations within counties is sufficient to address control strategies such as optimal ring culling. Here we instead focused on risk mapping and conversely found that small scale aggregation patterns can matter. Our study also differed from the set-up of Tildesley et al. in one important aspect. The result of Tildesley et al. were based on re-fitting the epidemic parameters to outbreak data on randomized locations. This approach is not possible for epidemiological modeling in populations without recent outbreaks like the U.S. In these instances, the transmission process has to be inferred from outbreaks in other countries [28].

Our results have important consequences for risk assessment for FMD and other livestock disease where local spread is a major pathway. The effect of realistic premises clustering introduced by FLAPS shows distinct spatial trends, with differences between configuration types most pronounced in large counties in the west. As such, it is not just the magnitude of risk that differs between FLAPS and randomized locations, but also the relative risk across the U.S. The use of randomized locations would substantially underestimate the risk of secondary infections in states like Arizona, California and New Mexico, likely due to relatively high spatial clustering of premises in these areas in the FLAPS configurations that is lost in the randomization process (Fig 2A–2C).

Epidemiological modeling offers powerful tools for contingency planning and may aid policy makers to evaluate risk and allocate resources for surveillance. Our study convincingly shows the potential importance of spatial patterns beyond randomization within counties. The lack of available data describing premises locations, inventories, and type have prompted computational methods to predict premises locations [23,29,30] and contact patterns [31,32] from limited available data. Other studies have provided methods to withhold identities of farmers when demography data is presented [33]. This approach is promising, but the effect on epidemic prediction remains unclear, and there is currently no expected change regarding the availability of detailed information on the spatial distributions of livestock premises in the U.S. Approaches such as FLAPS strive to include important realism based on landscape features, environmental conditions and anthropogenic factors associated with premises locations.

The problem of lacking data on spatial distributions of farm animal populations is not constrained to the U.S. Models similar to, or striving to solve the same issues as, FLAPS have been developed for various geographical regions and animal species such as cattle farms in Australia [34], poultry in China and the U.S. [29, 35], as well as various livestock in Europe and Africa [36, 37]. The existence of these models alone indicates that the problem with missing spatial data is also present in a larger context outside of the U.S, and although such models are not always developed mainly with epidemic modeling in mind, our study show that they can potentially play an important role for that in addition to other purposes.

Our results suggest that in the absence of true demographic data, methods such as FLAPS that realistically predict clustering patterns should be preferred over simple randomization of premises locations when modeling livestock diseases.

## Methods

### Premises populations

The analyses were based on two sets of premises populations, each with ten realizations of spatial configurations of the beef, dairy and feedlot premises populations of the 48 states and 3049 counties of the contiguous U.S. In the first set, each realization of spatial configuration was generated as a stochastic realization of the FLAPS model. Briefly, the FLAPS model disaggregates NASS county-level premises data by imputing missing data, predicting the geographic distribution of individual farms based on a probability surface, and simulating populations of animals on each farm [23]. The FLAPS probability surface was generated using logistic-regression and ten predictors that were fit to a national sample of presence or absence of livestock premises (*n* > 10,000 for each of the beef, dairy and feedlot populations). Verification indicates that FLAPS accurately imputes and simulates NASS premises data with a 0.03% error at the individual farm-to-county level. Thus, these configurations do not represent a true depiction of the U.S. premises population, but they account for realistic spatial configurations, including spatial clustering below the county level and are expected to accurately represent the number and size distribution of premises at the county level.

The second set of configurations consisted of ten realization of premises where locations were randomized within their respective county (Fig 1C). The exact number and type (i.e. beef, dairy, feedlot) of premises varied slightly between each FLAPS realization, and to obtain equivalent sets for comparison, we based the ten realizations of the randomized set on the FLAPS realizations to maintain all aspects of the premises population except for their location. We refer to the two sets of spatial premises distributions as FLAPS and random configurations, and each with ten realizations. It should be noted that the random configuration is only randomized in terms of their location within counties (i.e., we did not modify the estimated population sizes at each premise).

The ten FLAPS realizations and their corresponding random realizations included an average of 104,065,955 (103,930,597–104,177,356) calves and adult cattle distributed over an average of 812,703 premises (811,627–814,274). These numbers correspond well with the most up-to-date estimate of 103 million head [13] and the total number of premises in the categories *Farms with beef cows*, *Farms with milk cows* and *Farms with cattle on feed* presented in the 2012 U.S. NASS Census of Agriculture [14], which is currently the most recent census. However, compared to the category *Farms with Cattle and Calves* in the same publication, the FLAPS realizations underestimate the total number of premises by roughly 100,000. FLAPS simulates these three production types separately, but they were here combined into an all-encompassing depiction of the U.S. cattle premises demography.

The simulated populations of premises are available as supplementary material (S1 Dataset, S2 Dataset).

### Spatial clustering

As a measure of configuration-wide deviation from spatial homogeneity at different distance scales we calculated Ripley’s *K* [25]. Ripley’s *K* provides a statistic to determine if a set of points on a plane (e.g. a given spatial configuration of premises) follows a homogenous spatial distribution or not and is defined as

$$K\left(r\right)=\lambda^{-1}N^{-1}\sum_{i\in P}\sum_{\begin{array}{l} j\in P,\\ j\neq i \end{array}}I\left(d_{ij}<r\right).$$ (1)

where *λ* is the density of premises (premises per m^2^) within the landscape, **P** is the set of all *N* premises, *d*_*ij*_ is the distance between premises *i* and *j*, *r* is the distance scale of interest and *I* is a function that evaluates to one if *d*_*ij*_ is less than *r* and zero otherwise. Ripley’s K can be interpreted as the average density of points within the area *πr*^*2*^ of each focal point, averaged across all points in the landscape and expressed relative to the average density of the entire landscape. This landscape-level measure of *K* was calculated for each FLAPS realization and its randomized counterparts across a gradient of spatial scales *r = 10*^*0*^, *10*^*0*.*1*^, *…*, *10*^*1*.*9*^, *10*^*2*.*0*^ (Fig 2D). Additionally, in order to also quantify the degree of clustering of the premises within each county, we calculated a modified version of *K*, where instead of calculating the average relative density across all premises in the landscape, we calculated the average density across the premises within county *w*, denoting this subset of premises **P**_w_, as

$$K_{w}\left(r\right)=\lambda^{-1}N_{w}{}^{-1}\sum_{i\in P_{w}}\sum_{\begin{array}{l} j\in P\\ j\neq i \end{array}}I\left(d_{ij}<r\right).$$ (2)

### Epidemic models and kernels

We considered a premises-level Susceptible-Exposed-Infectious-Removed (SEIR) model framework commonly applied to fast spreading infectious diseases such as FMD [3,4,38–40]. In this framework, the rate of transmission, *λ*_*ij*_, between infectious premises *i* and susceptible premises *j* over time period *δt* (here expressed in days) is determined by the transmissibility and susceptibility of the premises as well as a kernel function, *H*, describing how the transmission rate varies with between-premises distance *d*_*ij*_ as

$$\lambda_{ij}=S\left(n_{j}\right)T\left(n_{i}\right)H\left(d_{ij}\right)\delta t.$$ (3)

Functions *T* and *S* scale the transmissibility and susceptibility respectively based on the premises sizes of *n*_*i*_ and *n*_*j*_, respectively. Given this rate, the probability that transmission occurs between infectious premises *i* and susceptible premises *j* can be discretized over the time interval of *δt* days by:

$$p_{ij}=1-e^{-\lambda_{ij}}.$$ (4)

Following infection, the premises enters the exposed (E) class, a latent stage where it is infected but not yet infectious. After the exposed period, the premises transition into the infectious (I) class, and its entire cattle population is considered infectious. This simplifying assumption is often justified by the high infectiousness and rapid within-herd spread of FMD-like diseases [19,41]. After the infectious period, the premises transitions into the removed (R) class, where it can neither infect other premises nor become infected.

Due to the lack of recent FMD outbreaks in the U.S., there are no outbreak data to fit models to. Therefore, our approach was to implement a set of published kernels that encompasses a substantial range of assumptions, thereby investigating the effect of spatial clustering under a range of plausible scenarios. We identified three kernel models available from the literature that model the transmission process with the general form of Eq (3) but implement different functional forms and/or parameterizations of *S*, *T*, and *H*. They also vary in infectious and latency periods. Parameters and functional forms are presented in Table 3, and Fig 3 shows the shapes of the kernel functions *H* (panel A) and the relationship between herd size and *S* and *T* (panel B).

The first kernel model was obtained from Brand et al. [19]. This study implemented several similar kernels, and we selected the one with the largest spatial range of *H* to obtain a case of transmission over large distances.

The second kernel was obtained from Hayama et al. [3] and was developed for and found to provide a good fit to the FMD outbreak of Japan in 2010. We made one alteration to fit with the general framework of our study. In the original model by Hayama et al. [3] infectious premises did not recover after a fixed time period but were considered infectious until culled, and the time between infection and culling is expressed as a function of the amount of simultaneously infected premises. To allow for an analytical solution for *R*_*0*_, we instead set the infectious period to a fixed number of 16 days, which is an intermediate value of the range used in [3].

The third kernel was used by Tildesley et al. [24], who implemented a kernel model fitted to the 2001 United Kingdom (U.K.) FMD and modified it to model FMD outbreaks in Pennsylvania. The kernel *H* is non-parametric and is defined as a look-up table with infection risks at binned distances, derived from contact tracing during the U.K. 2001 outbreak. To fit with the larger distances of the U.S., Tildesley et al. [24] added a parameter to increase the kernel’s relative width in relation to the U.K. kernel. We used the same parameter in this study, choosing a value (*β = 4*.*0*) from the range analyzed in the original study that yielded a width falling in between the Brand and Hayama kernels (Fig 3A). As stated in the work by Tildesley et al. [24], increasing the width of the kernel in this manner also increases the total magnitude of transmission, so in order to preserve the original volume under the function, the resulting kernel was scaled by an additional parameter (*α = 0*.*0625*).

**Table 3:** Kernel parameters and functional forms. Infectious period refers to the number of days that an infected premises is infectious (i.e. can transmit the pathogen to another premises), and exposed period refers to the number of days between date of transmission and becoming infectious. For the Tildesley kernel, *H*_*UK*_ represents the nonparametric kernel fitted to the U.K. 2001 FMD outbreak [4].

Kernel	Susceptibility	Transmissibility	Infectious period (days)	Exposed period (days)	Reference
*Brand*	*S*(*n*) = *n*^0.2^	*T*(*n*) = *n*^0.2^	5	4	[19]
*Hayama*	*S*(*n*) = ln *n*	*T*(*n*) = ln *n*	16	2	[3]
*Tildesley*	*S*(*n*) = 5.7*n*^0.41^	*T*(*n*) = 0.00082*n*^0.42^	5	4	[24]
	Kernel functional form		α	β	γ
*Brand*	$H\left(d_{ij}\right)=0.115\alpha_{\gamma }\left(\beta \right){\left(\beta^{2}+d_{ij}^{2}\right)}^{-\gamma /2}$		190.99	20.0	5.0
*Hayama*	$H\left(d_{ij}\right)=\alpha {\left(1+\frac{d_{ij}}{\beta }\right)}^{-\gamma }$		7.4e-4	0.58	2.47
*Tildesley*	$H\left(d_{ij}\right)=\alpha H_{UK}\left(\frac{d_{ij}}{\beta }\right)$		0.0625	4.0	-

### Computing the Basic reproductive number, R_0_

The basic reproductive number, *R*_*0*_, is the expected number of secondary cases following infection of premises *i* during its infectious period in a population of entirely susceptible premises [42]. To obtain a premises level *R*_*0*_, we computed the sum of the pairwise probabilities of *i* infecting any other premises *j* belonging to the set of all premises **P**. We denote *R*_*0*_ of premises *i*, *R*_*i*_, given by

$$R_{i}=\sum_{\begin{array}{l} j\in P,\\ j\neq i \end{array}}p_{ij},$$ (5)

where *p*_*ij*_ is given by Eq (4) exchanging *δt* for the infectious period of the kernel used. The premises level *R*_*i*_ was calculated for every premises in each of the 20 realizations. In order to identify spatial differences in expected outbreak risk levels, we calculated the geometric mean *R*_*i*_, for each county *w*, across all ten FLAPS or randomized realizations. Thus, we obtained a single measure of the basic reproductive number of the pathogen at the county-level for each configuration, which we denoted ${\hat{R}}_{w}$. We chose the geometric mean over the arithmetic mean as the epidemic process is better described as a multiplicative rather than an additive process.

### Simulations

For each of the three kernels, numerical simulations were performed with 1000 replicates per county and landscape realization, totaling 30,490,000 outbreak simulations per landscape type (FLAPS or randomized) and kernel. Each simulated outbreak was seeded with a single infectious premises, which was picked randomly within the focal county. The simulations had a temporal resolution of *δt* = 1 day and were run until they either died out or (to keep simulations time at a manageable level) reached 100 cumulative infected premises. This cutoff was assumed to suffice to evaluate the risk of large outbreaks based on the typically bimodal behavior during the early stage of the simulations; either the outbreak takes off and spreads to a large number of premises, or it dies out soon after seeding. To verify that 100 premises was a reliable proxy for even larger outbreaks, we also ran equivalent outbreak simulations that were allowed to continue until they died out, regardless of number of infected premises, albeit only seeding in a subset of counties. This stratified subset consisted of one county per contiguous state, selected as the county with the median number of premises for the state. The initial simulations resulted in very few outbreaks taking off, making comparison between configurations difficult due to lack of data. Therefore, we also performed a set of simulations where the transmission rate (*λ*_*ij*_) between the premises was increased by a factor of five. This resulted in substantially more outbreaks taking off, making it possible to assess differences between randomized and FLAPS configurations.

The simulations were performed using the FMD simulation algorithm published by Sellman et al. [43] (where the C++ code is made available), in which the population of premises is subdivided into a grid structure that enables a reduction in the computational complexity of the numerical simulation. This approach speeds up the calculations without adding any approximations beyond the temporal discretization of the epidemic process, which was already present in the implemented models.

To test the effect of clustering and configuration on the risk of obtaining large outbreaks, we performed a logistic regression for each of the three kernels with the Bernoulli trial of reaching 100 cumulative infected premises or not as the dependent variable. For explanatory variables, referred to in italics, we used the logarithms of the average premises size and the number of premises in the county in which the outbreak was seeded (*avg*. *prem*. *size*, and *N*. *prem*.), the logarithm of the premises size of the seeded premises (*seeded size*) and a binary variable indicating if the configuration used was FLAPS or randomized (*landscape configuration)*. Additionally, the clustering of the county of the seeded premises was also included as an explanatory variable (*clustering*). Because the effect of clustering at various spatial scales on outbreaks will depend on the choice of kernel, this clustering was calculated with the distance at which the respective kernels had fallen to 0.05 of the value evaluated at *d = 0*, or in other words, the distance at which the magnitude of the kernel had fallen by 95%. We denote this distance $\hat{r}$, and determined it to be at 30.4, 1.4 and 6.6 km for *H*_*Brand*_, *H*_*Hayama*_ and *H*_*Tildesley*_, respectively, and we denote the county-level Ripley’s *K* evaluated at this distance as $K_{w}^{\hat{r}}$. From this we derived the scale-independent, variance stabilized measure $L_{w}^{\hat{r}}=\sqrt{K_{w}^{\hat{r}}\pi^{-1}}-\hat{r}$ which is what was used in the regression analysis as a measure of county-level clustering [44]. Finally, to capture the residual variance between landscape realizations, a binary variable indicating which of the ten realizations that was used was also included in the model as a random effect (*real*. *1–10*). All the explanatory variables were standardized to have a mean of zero and unit variance prior to the analyses.

With 60,980,000 seeded outbreaks per kernel, assessing significance is not meaningful [45]. Instead, we evaluated the importance of each variable by first performing regression analyses with the full set of explanatory variables included and, subsequently, five additional regression analyses, each with one parameter removed. In order to compare the resulting models we calculated Tjur’s *coefficient of discrimination* (*CoD*), a measure of goodness of fit suitable for logistic regression [46]. For logistic regression, the *CoD* is an analogue of the ubiquitous coefficient of determination (*R*^*2*^) of linear regression models and ranges from 0 to 1. The relative difference in *CoD* between the full and the reduced model, denoted Δ*CoD* = (*CoD*_*reduced*_−*CoD*_*full*_)/*CoD*_*full*_, quantifies how goodness of fit is reduced when the focal parameter is excluded. Thus, *ΔD* provides an estimate of the importance of the removed parameter to explain the risk of a large outbreak.

To determine the effect clustering had on the speed with which the outbreaks developed, we performed two linear regression analyses for each kernel using the same set of predictors as for the logistic regression but with time step at which 10 and 100 infected premises were reached as dependent variable respectively. These results are presented in the supplementary material S4 Table.

Analyses were performed using C++ and Python with NumPy [47], pandas [48], scikit-learn [49] and matplotlib [50].

## Supporting information

S1 FigFrequency and spatial distributions of minimum premises-level *R*_*0*_ for each county, *R*_*w*,*min*_ and relative difference in *R*_*w*,*min*_ between FLAPS and random configurations.Minimum *R*_*i*_ across the premises of each county *w* for FLAPS ($R_{w,\min}^{FLAPS}$) and randomized ($R_{w,\min}^{Rand.}$, dashed) and configurations (left). Frequency distribution of the proportional difference in *R*_*w*,min_ between configurations shown by the black line in histograms (left) and its spatial distribution is illustrated by the maps. The proportional difference shown by the black histogram is independent of the increase applied to the transmission rate.(TIFF)

S2 FigFrequency and spatial distributions of maximum premises-level *R*_*0*_ for each county, *R*_*w*,*max*_ and relative difference in *R*_*w*,*max*_ between FLAPS and random configurations.Maximum *R*_*i*_ across the premises of each county *w* for FLAPS ($R_{w,\max}^{FLAPS}$) and randomized ($R_{w,\max}^{Rand.}$, dashed) and configurations (left). Frequency distribution of the proportional difference in *R*_*w*,max_ between configurations shown by the black line in histograms (left) and its spatial distribution is illustrated by the maps. The proportional difference shown by the black histogram is independent of the increase applied to the transmission rate.(TIFF)

S3 FigR_0_ difference vs county area.Proportional change in ${\hat{R}}_{w}^{FLAPS}$ compared to ${\hat{R}}_{w}^{Rand.}$ against county area showing that larger counties see a bigger change in ${\hat{R}}_{w}$. *R*^*2*^*-*values for the linear regression are Brand (A): 0.56, *p <0*.*01;* Hayama (B): 0.44, *p<0*.*01;* Tildesley (C): 0.52, *p<0*.*01*.(TIFF)

S4 FigBetween-realization difference in *R*_*w*_.Histograms showing for each county the largest difference in ${\hat{R}}_{w}$ from the median ${\hat{R}}_{w}$ over all ten FLAPS realizations.(TIFF)

S5 Fig Relationship between the proportion of simulations reaching 100 vs 1,000 and 10,000 cumulative infected premises. Each point represents results from 10,000 simulations of outbreaks starting in one of 48 counties, each being the county with the median number of premises within its state. Most points are located on the diagonal line, which is expected if an outbreak that reaches 100 infected premises also reach 1,000 or 10,000 infected premises respectively.
(TIFF)

S6 FigRelationship between configuration effect on geometric mean county level reproductive rate ${\hat{R}}_{w}$ and proportion of simulations reaching outbreak sizes, *l*.The figure shows the ratio between the proportion of simulations reaching *l =* 2, 10 or 100 cumulative infected premises in simulations with FLAPS configuration and randomized configurations ($prop_{l}^{FLAPS}/prop_{l}^{Rand.}$) plotted against the corresponding county ratio of reproductive number (${\hat{R}}_{w}^{FLAPS}/{\hat{R}}_{w}^{Rand.}$). Panels show the results for the three different kernels (Brand, Hayama and Tildesley) with a linear regression line fit to the log transformed data, *R*^*2*^ and *p* values in legend.(TIFF)

S7 Fig Simulation results. **Transmissibility x5, random configuration.** Color indicates the proportion of simulated outbreaks that reach 100 infected premises or more out of 10,000 replicates if outbreak starts in this county. Grey indicates that no outbreak started in the county reached 100 infected premises.
(TIFF)

S8 Fig Simulation results. **Transmissibility x5, FLAPS configuration.** Color indicates the proportion of simulated outbreaks that reach 100 infected premises or more out of 10,000 replicates if outbreak starts in this county. Grey indicates that no outbreak started in the county reached 100 infected premises.
(TIFF)

S9 Fig Differences in probability of large outbreaks. **Transmissibility x1.** County-level proportional change in number of replicates that reached 100 infected premises when using FLAPS compared to randomized configurations. Grey indicates counties where no replicate reached 100 infected in either FLAPS simulations or randomized simulations or both. Results are based on original kernel parameterizations, i.e. without five-fold increase in transmissibility.
(TIFF)

S10 Fig Simulation results. **Transmissibility x1, random configuration.** Color indicates the proportion of simulated outbreaks that reach 100 infected premises or more out of 10,000 replicates if outbreak starts in this county. Grey indicates that no outbreak started in the county reached 100 infected premises.
(TIFF)

S11 Fig Simulation results. **Transmissibility x1, FLAPS configuration.** Color indicates the proportion of simulated outbreaks that reach 100 infected premises or more out of 10,000 replicates if outbreak starts in this county. Grey indicates that no outbreak started in the county reached 100 infected premises.
(TIFF)

S1 Table The proportion of simulated outbreaks that reached 10,000 infected premises. Each combination of kernel, transmissibility scale factor and spatial premises distribution is based on 480,000 seeded outbreaks.
(PDF)

S2 TableLogistic regression, simulation transmissibility x1.Coefficient of discrimination (*CoD*), relative change in *CoD* (*ΔCoD)* and estimated coefficients of the predictors of logistic regression models predicting the probability of reaching 100 infected premises. The predictors are the county-level clustering at a spatial scale relevant for the kernel, $K_{w}^{\hat{r}}$ *(clustering)*, the type of configuration (random or FLAPS, *landscape conf*.), the logarithm of the average premises size in the seeded county (*avg*. *prem*. *size*), the logarithm of the number of premises in the seeded county (*N*. *prem*.) and the logarithm of the premises size of the seeded premises, (*seeded size*).(PDF)

S3 TableLogistic regression, simulation transmissibility x5.Coefficient of discrimination (*CoD*), relative change in *CoD* (*ΔCoD)* and estimated coefficients of the predictors of logistic regression models predicting the probability of reaching 100 infected premises. The predictors are the county-level clustering at a spatial scale relevant for the kernel, $K_{w}^{\hat{r}}$ *(clustering)*, the type of configuration (random or FLAPS, *landscape conf*.), the logarithm of the average premises size in the seeded county (*avg*. *prem*. *size*), the logarithm of the number of premises in the seeded county (*N*. *prem*.) and the logarithm of the premises size of the seeded premises, (*seeded size*).(PDF)

S4 Table Explanatory power of predictors for time steps passed before reaching 10 and 100 infected premises. Relative correlation coefficients from linear regression of independent variables vs dependent variable *number of time steps to reach l infected premises* where *l* is 10, or 100. The same set of independent variables were used as in the logistic regression. For each kernel, only the subset of simulations that reached *L* were used in the regression and only the results from the simulations based on transmissibility x5 were used. The number of time steps (days) required to reach 100 infected premises was highly affected by clustering. High clustering in the seeded county generally led to faster development of the outbreak, and a similar, although less substantial, relationship with landscape configuration is seen. We point out that one caveat to this analysis is that it does not take into account the higher frequency with which the simulations with FLAPS landscape configurations reached *l*.
(PDF)

S1 Dataset FLAPS premises populations. Ten simulated spatial distributions of US cattle premises, simulated using FLAPS.
(ZIP)

S2 Dataset Randomized premises populations. Same as the FLAPS premises distributions, but the premises’ coordinates have been randomized within the counties they are found in.
(ZIP)

S1 Appendix Maximal outbreak size. (PDF)
