# Supplementary figures and images for: Realistic assumptions about spatial locations and clustering of premises matter for models of foot-and-mouth disease spread in the United States
Source: PLoS Comput Biol. 2020 Feb 20;16(2):e1007641. doi: 10.1371/journal.pcbi.1007641 (PMC7053778; doi:10.1371/journal.pcbi.1007641)

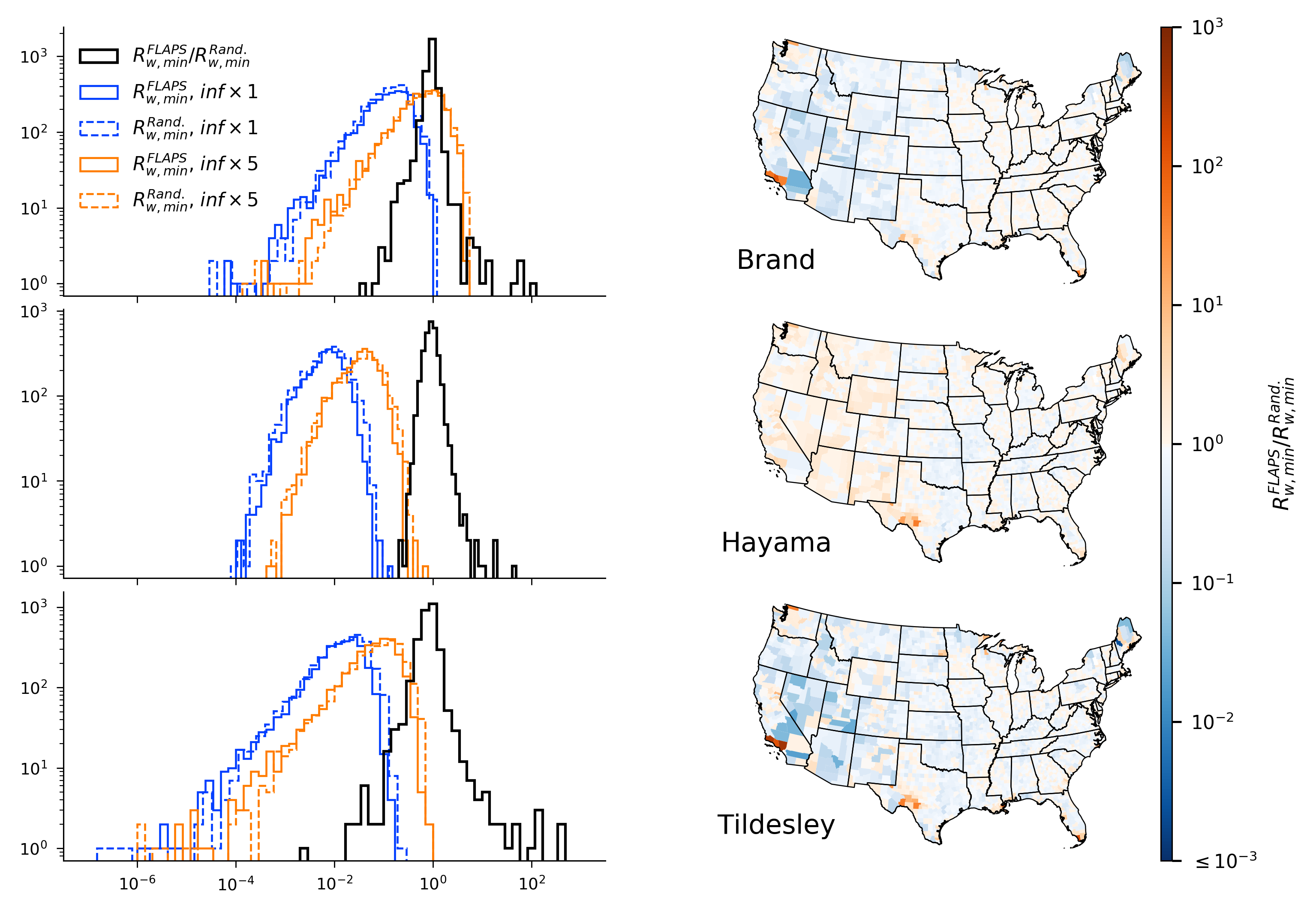

Supplement: S1 Fig — Minimum Ri across the premises of each county w for FLAPS (Rw,minFLAPS) and randomized (Rw,minRand., dashed) and configurations (left). Frequency distribution of the proportional difference in Rw,min between configurations shown by the black line in histograms (left) and its spatial distribution is illustrated by the maps. The proportional difference shown by the black histogram is independent of the increase applied to the transmission rate. (TIFF) [file pcbi.1007641.s001.tiff]

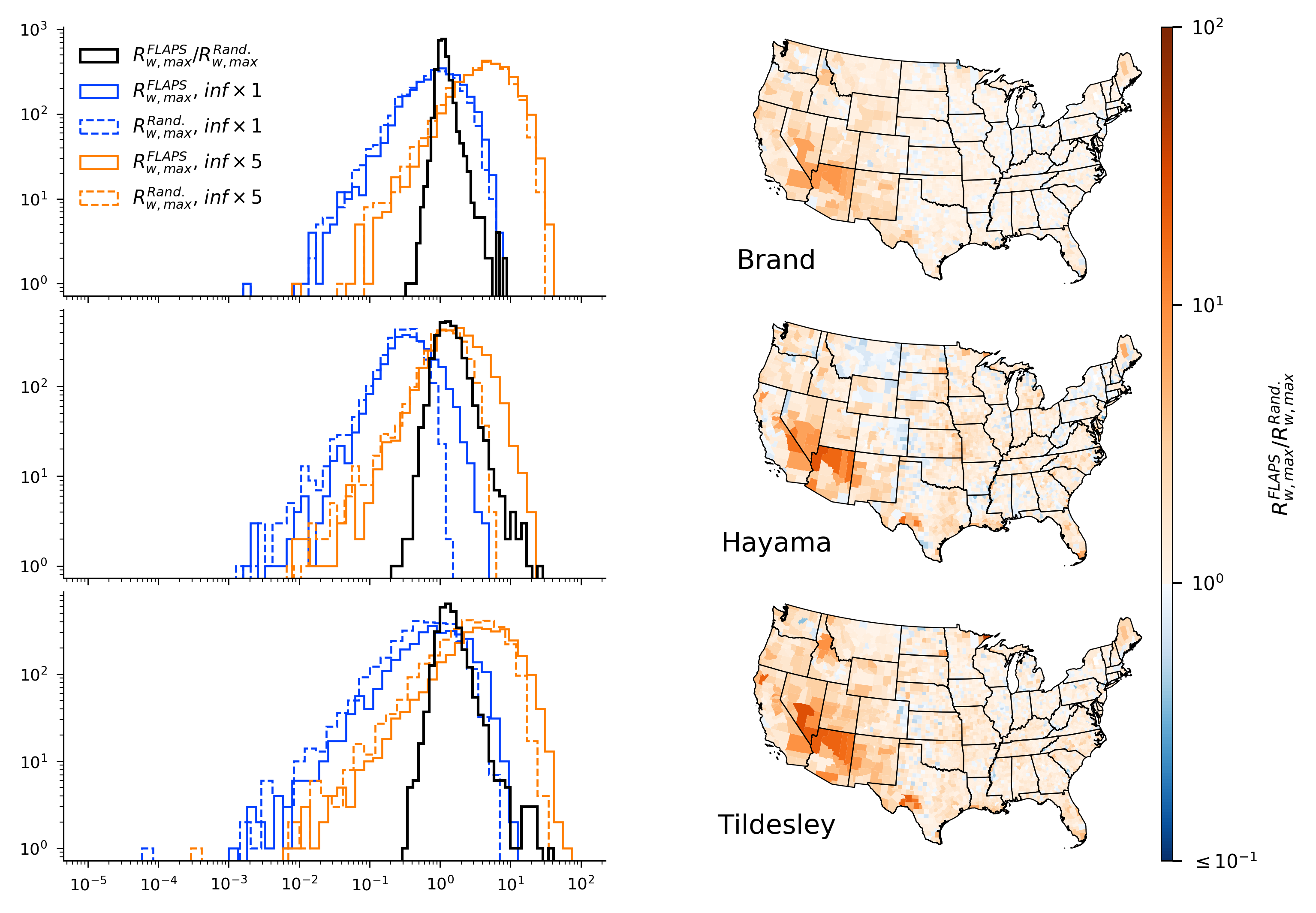

Supplement: S2 Fig — Maximum Ri across the premises of each county w for FLAPS (Rw,maxFLAPS) and randomized (Rw,maxRand., dashed) and configurations (left). Frequency distribution of the proportional difference in Rw,max between configurations shown by the black line in histograms (left) and its spatial distribution is illustrated by the maps. The proportional difference shown by the black histogram is independent of the increase applied to the transmission rate. (TIFF) [file pcbi.1007641.s002.tiff]

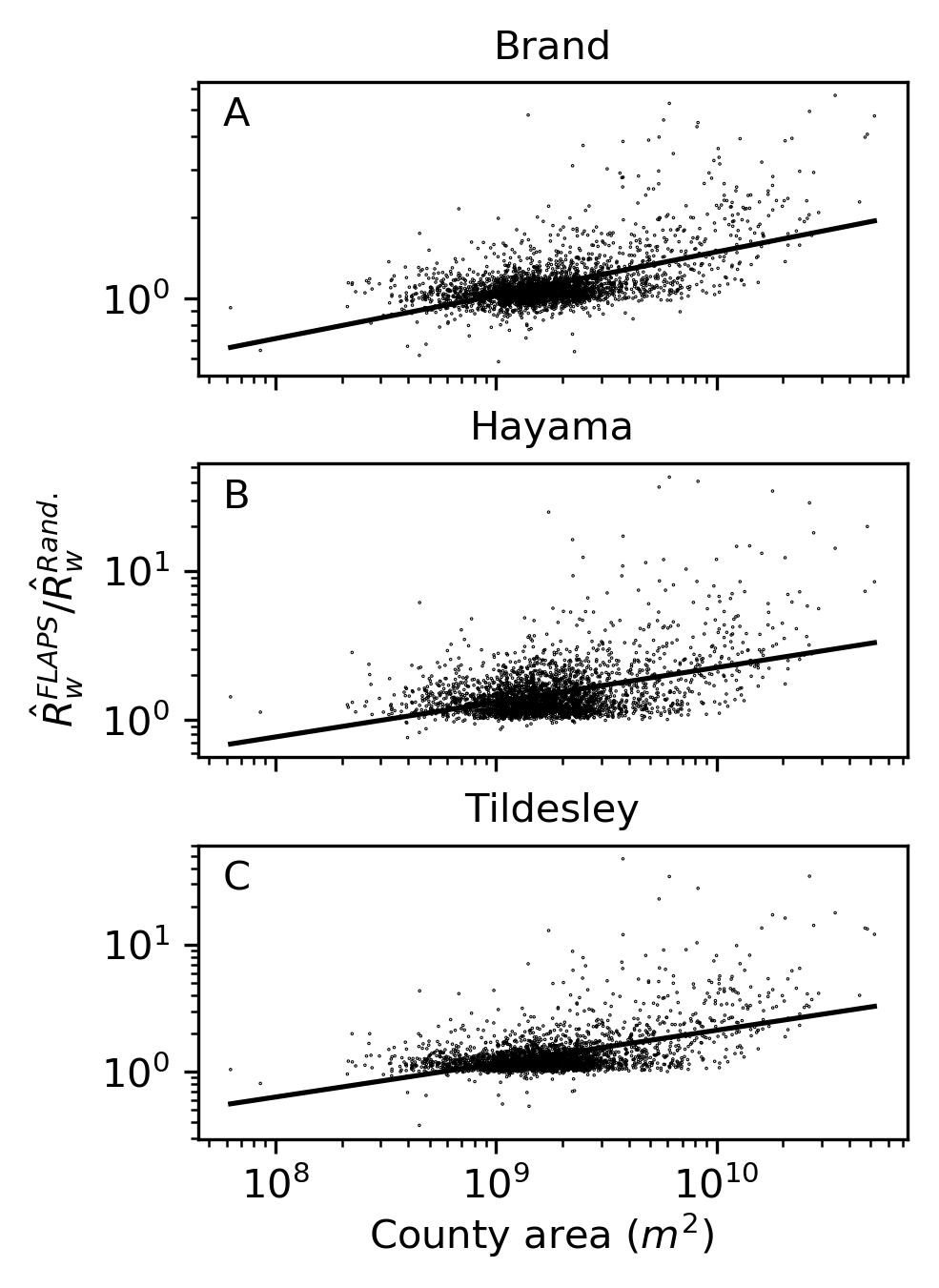

Supplement: S3 Fig — Proportional change in R^wFLAPS compared to R^wRand. against county area showing that larger counties see a bigger change in R^w. R2-values for the linear regression are Brand (A): 0.56, p <0.01; Hayama (B): 0.44, p<0.01; Tildesley (C): 0.52, p<0.01. (TIFF) [file pcbi.1007641.s003.tiff]

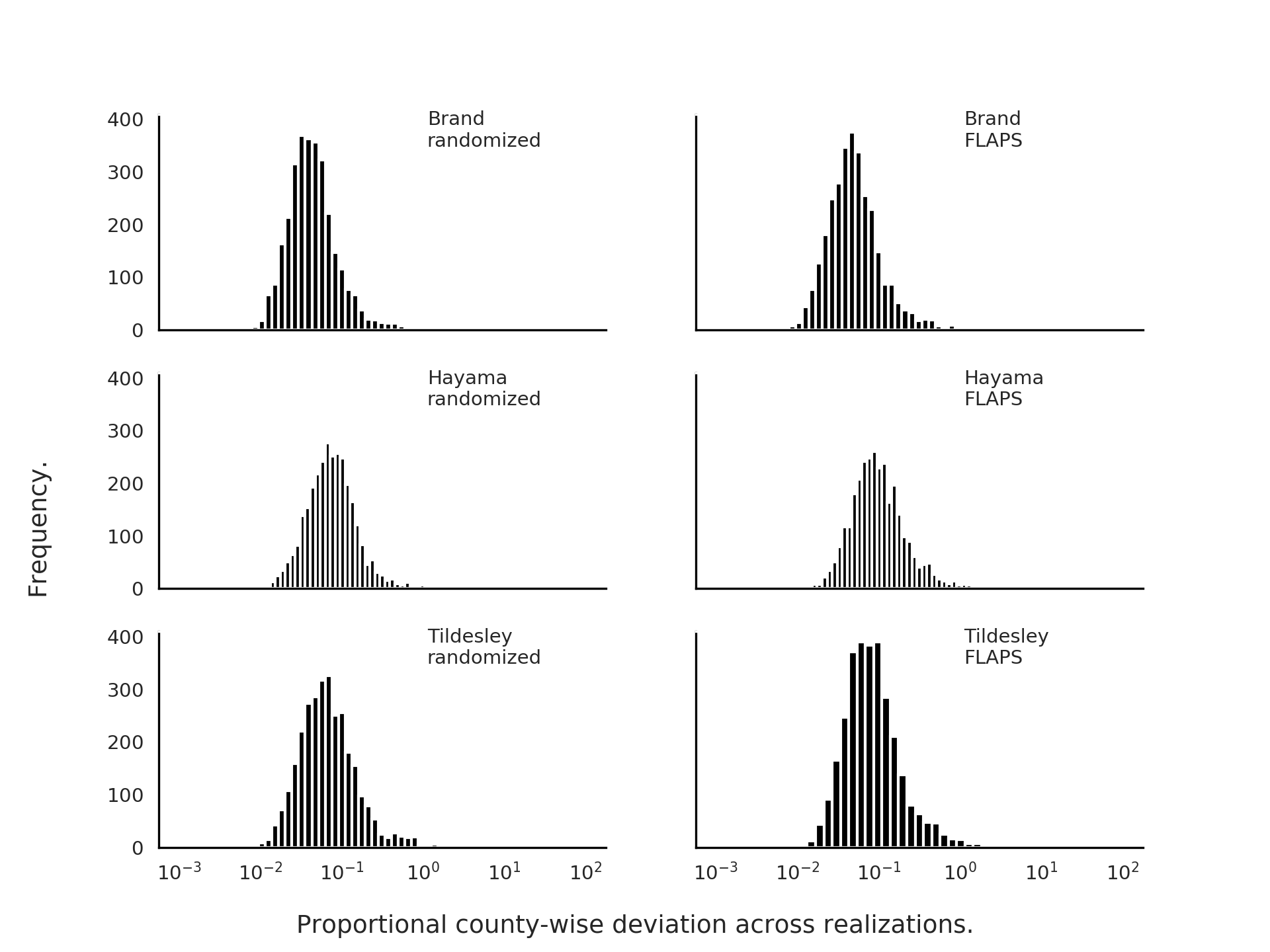

Supplement: S4 Fig — Histograms showing for each county the largest difference in R^w from the median R^w over all ten FLAPS realizations. (TIFF) [file pcbi.1007641.s004.tiff]

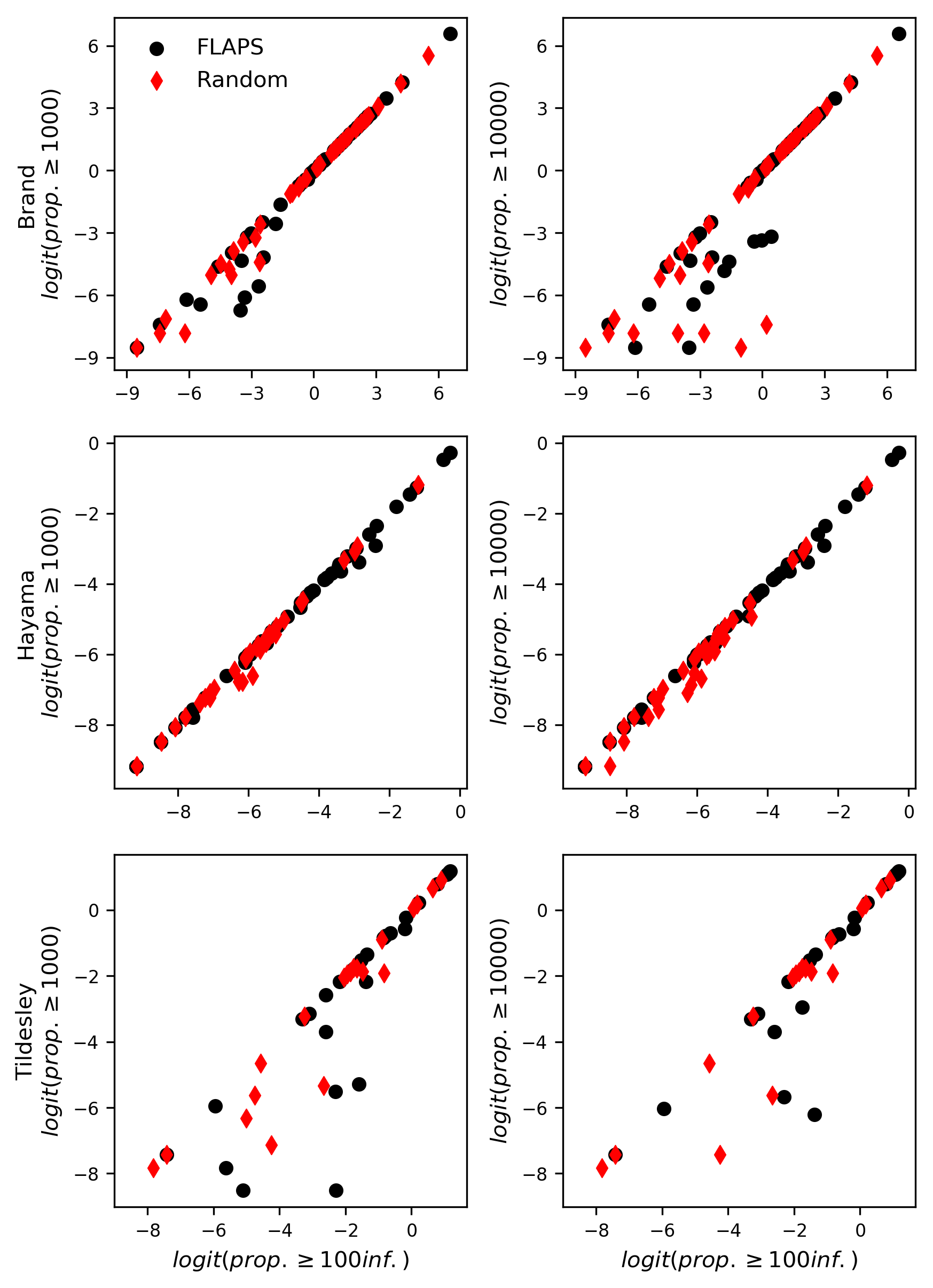

Supplement: S5 Fig — Each point represents results from 10,000 simulations of outbreaks starting in one of 48 counties, each being the county with the median number of premises within its state. Most points are located on the diagonal line, which is expected if an outbreak that reaches 100 infected premises also reach 1,000 or 10,000 infected premises respectively. (TIFF) [file pcbi.1007641.s005.tiff]

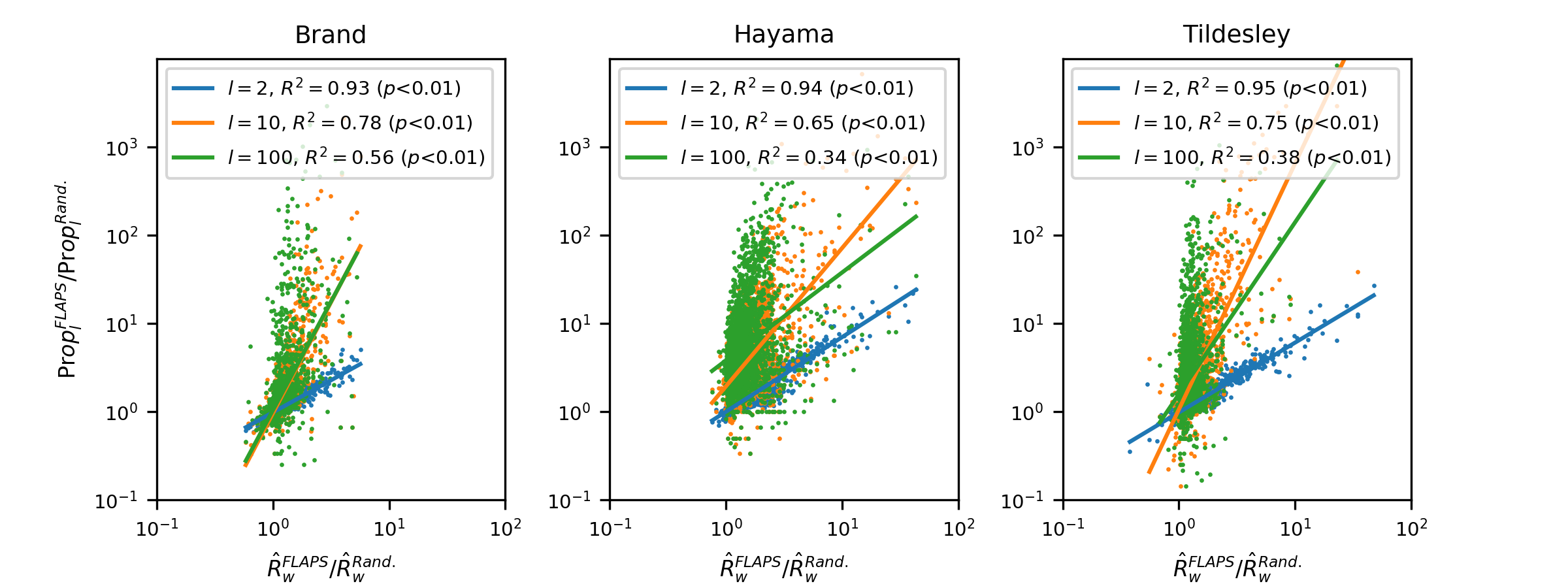

Supplement: S6 Fig — The figure shows the ratio between the proportion of simulations reaching l = 2, 10 or 100 cumulative infected premises in simulations with FLAPS configuration and randomized configurations (proplFLAPS/proplRand.) plotted against the corresponding county ratio of reproductive number (R^wFLAPS/R^wRand.). Panels show the results for the three different kernels (Brand, Hayama and Tildesley) with a linear regression line fit to the log transformed data, R2 and p values in legend. (TIFF) [file pcbi.1007641.s006.tiff]

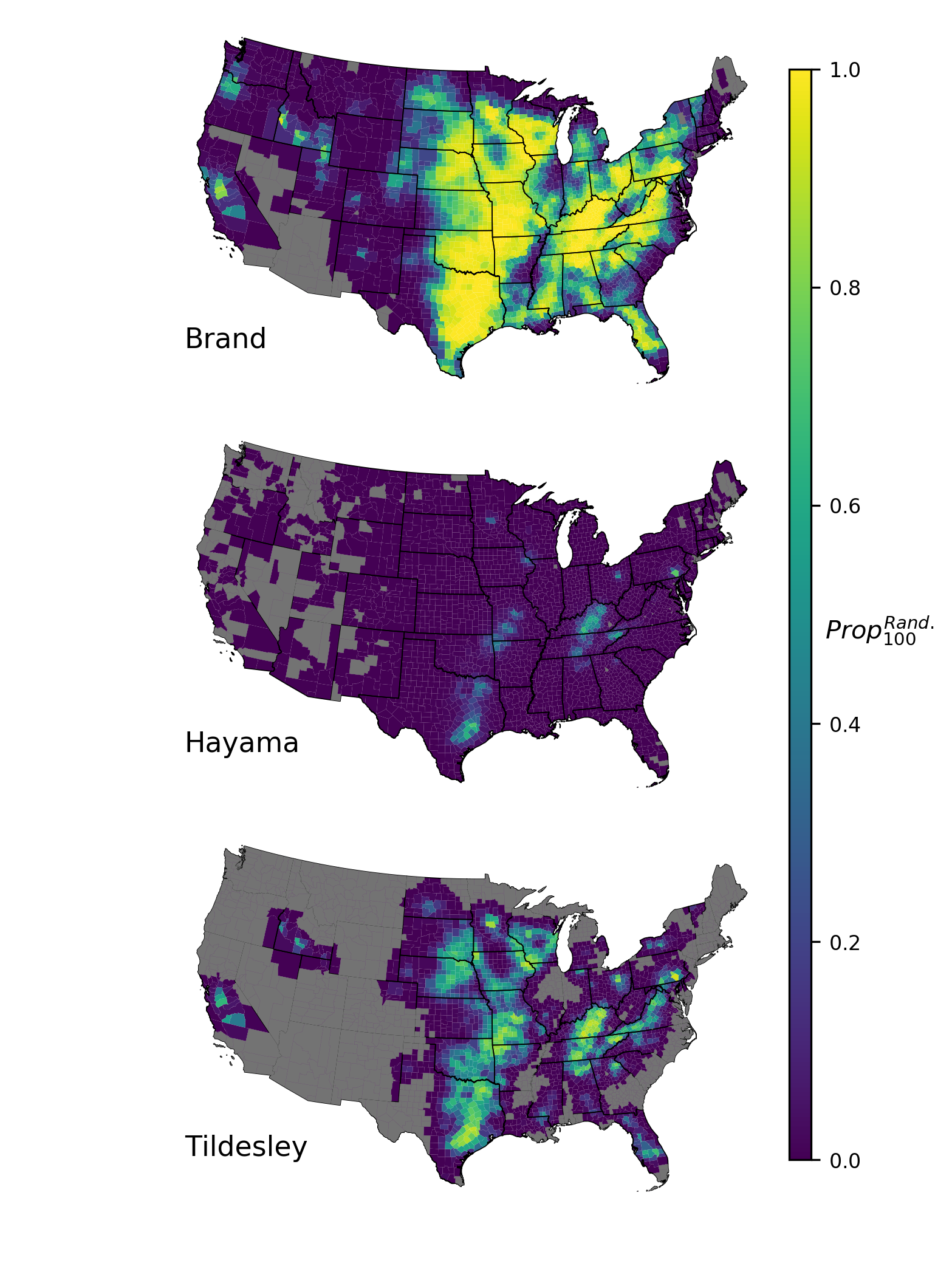

Supplement: S7 Fig — Transmissibility x5, random configuration. Color indicates the proportion of simulated outbreaks that reach 100 infected premises or more out of 10,000 replicates if outbreak starts in this county. Grey indicates that no outbreak started in the county reached 100 infected premises. (TIFF) [file pcbi.1007641.s007.tiff]

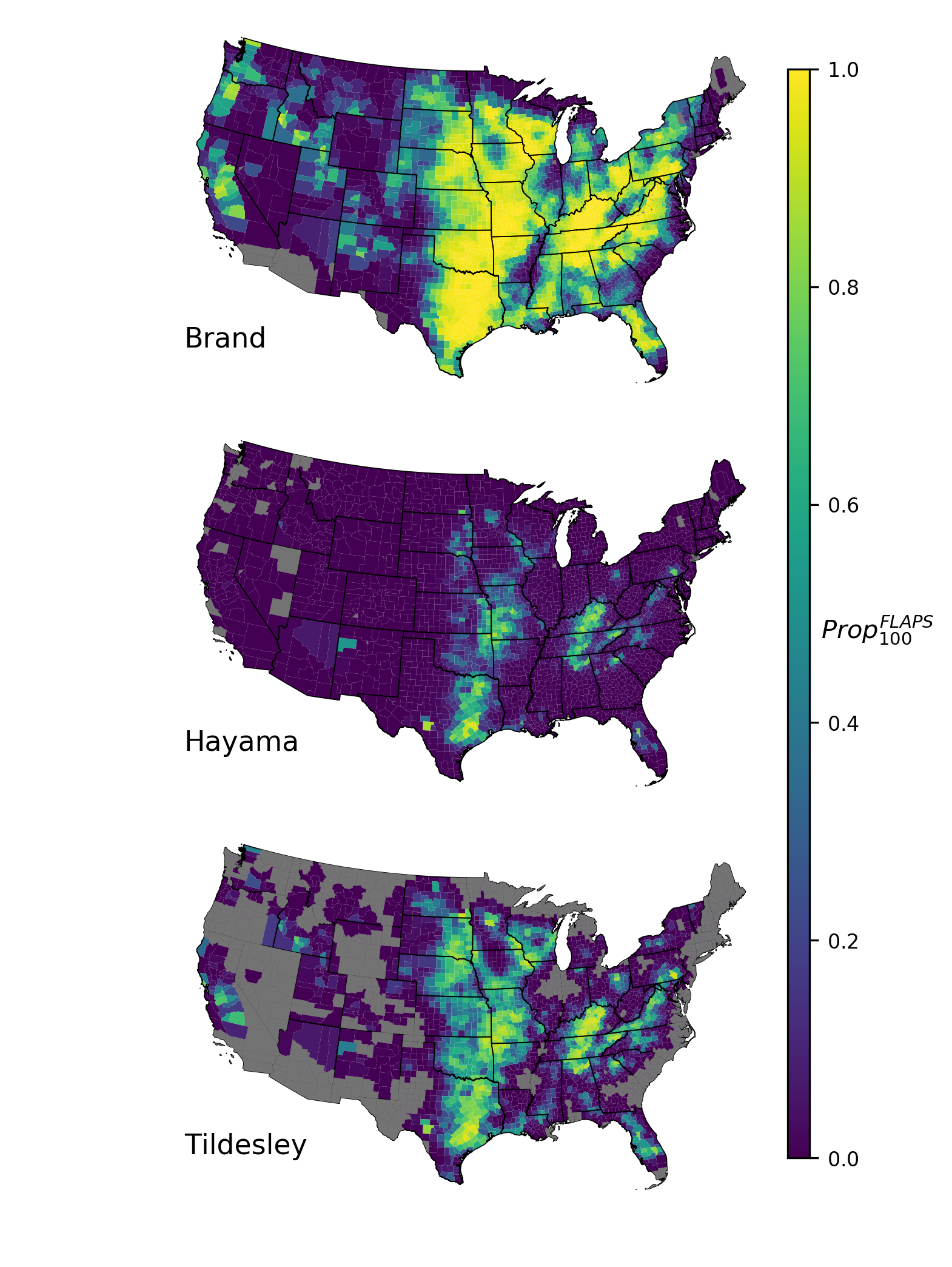

Supplement: S8 Fig — Transmissibility x5, FLAPS configuration. Color indicates the proportion of simulated outbreaks that reach 100 infected premises or more out of 10,000 replicates if outbreak starts in this county. Grey indicates that no outbreak started in the county reached 100 infected premises. (TIFF) [file pcbi.1007641.s008.tiff]

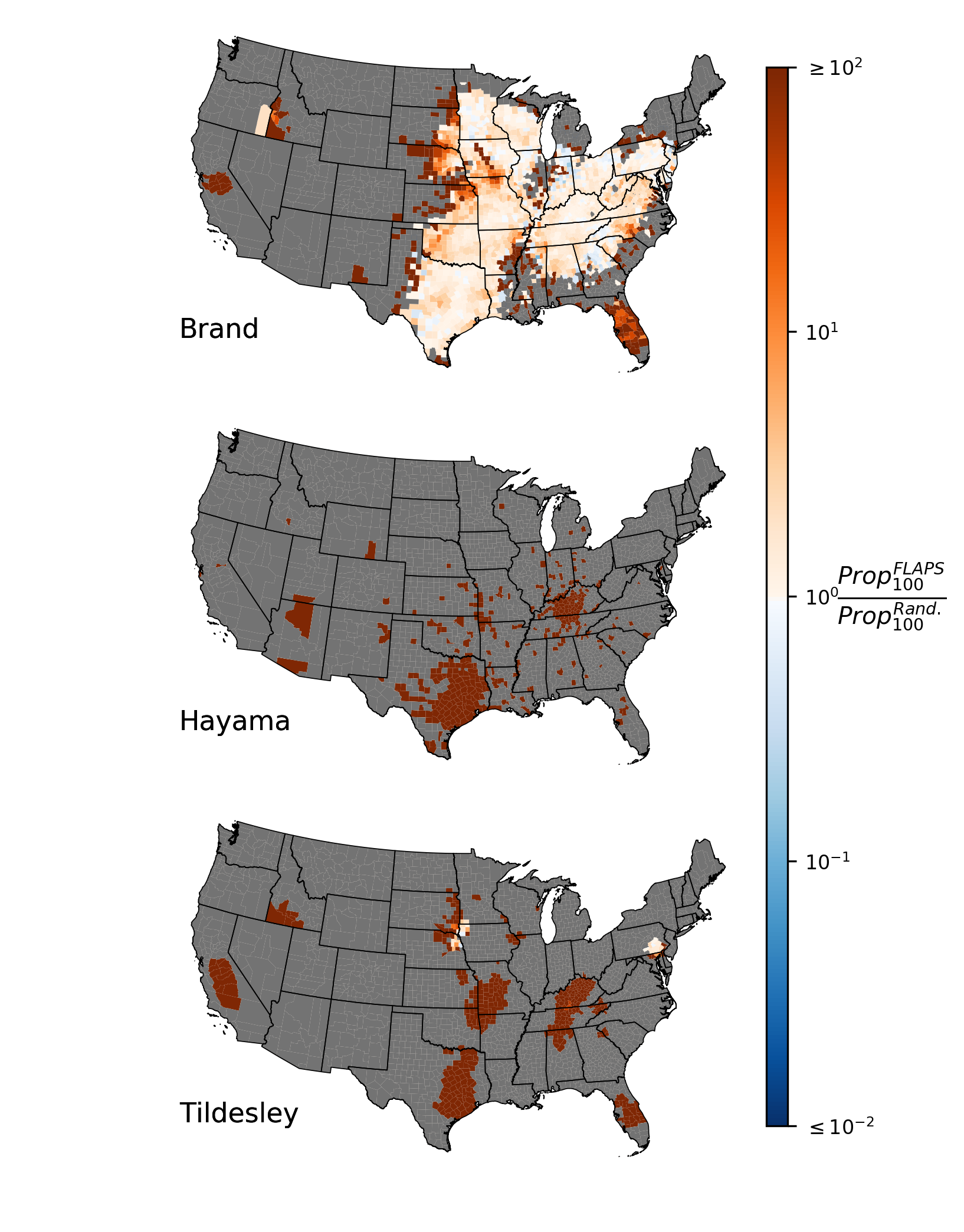

Supplement: S9 Fig — Transmissibility x1. County-level proportional change in number of replicates that reached 100 infected premises when using FLAPS compared to randomized configurations. Grey indicates counties where no replicate reached 100 infected in either FLAPS simulations or randomized simulations or both. Results are based on original kernel parameterizations, i.e. without five-fold increase in transmissibility. (TIFF) [file pcbi.1007641.s009.tiff]

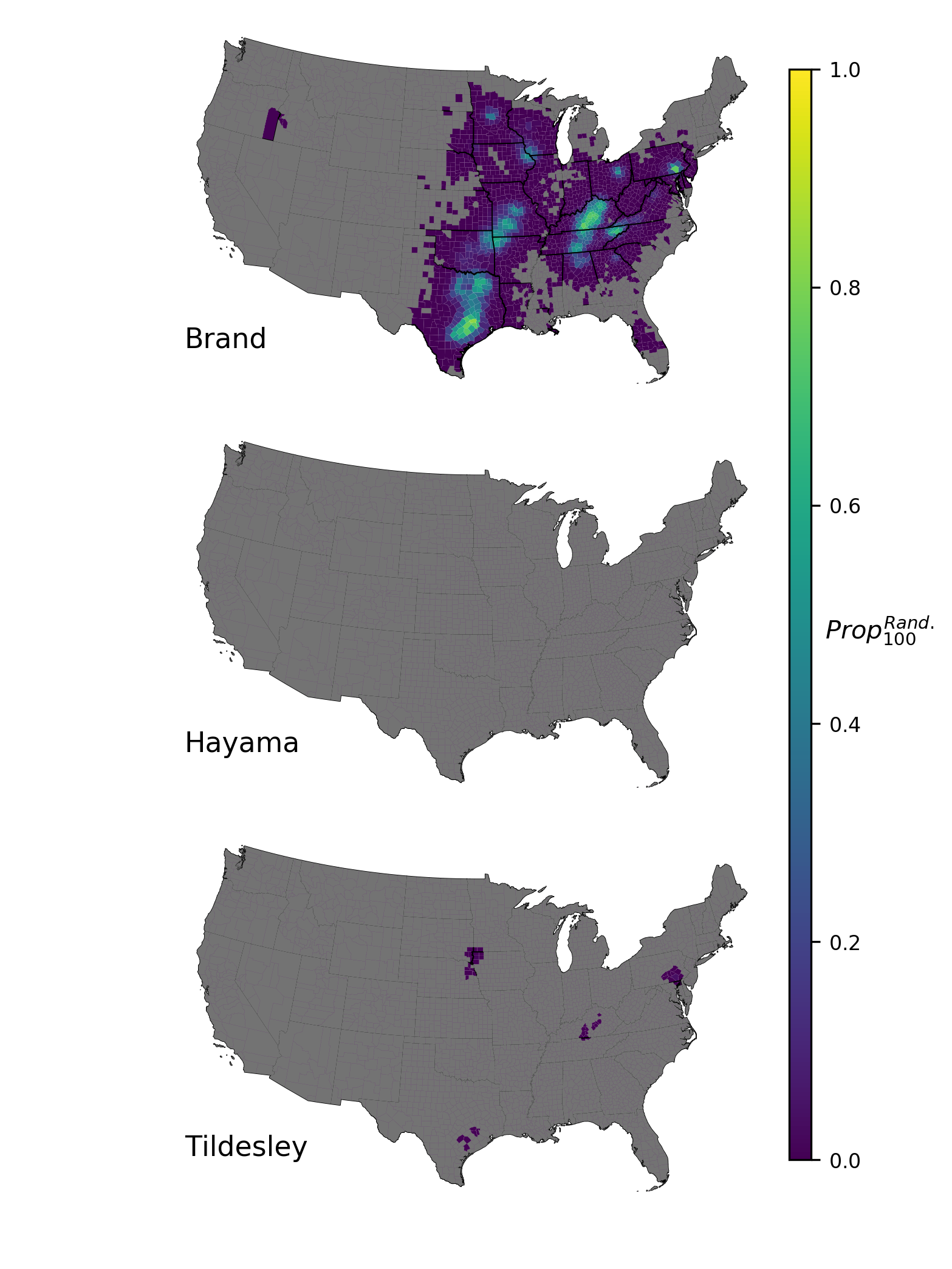

Supplement: S10 Fig — Transmissibility x1, random configuration. Color indicates the proportion of simulated outbreaks that reach 100 infected premises or more out of 10,000 replicates if outbreak starts in this county. Grey indicates that no outbreak started in the county reached 100 infected premises. (TIFF) [file pcbi.1007641.s010.tiff]

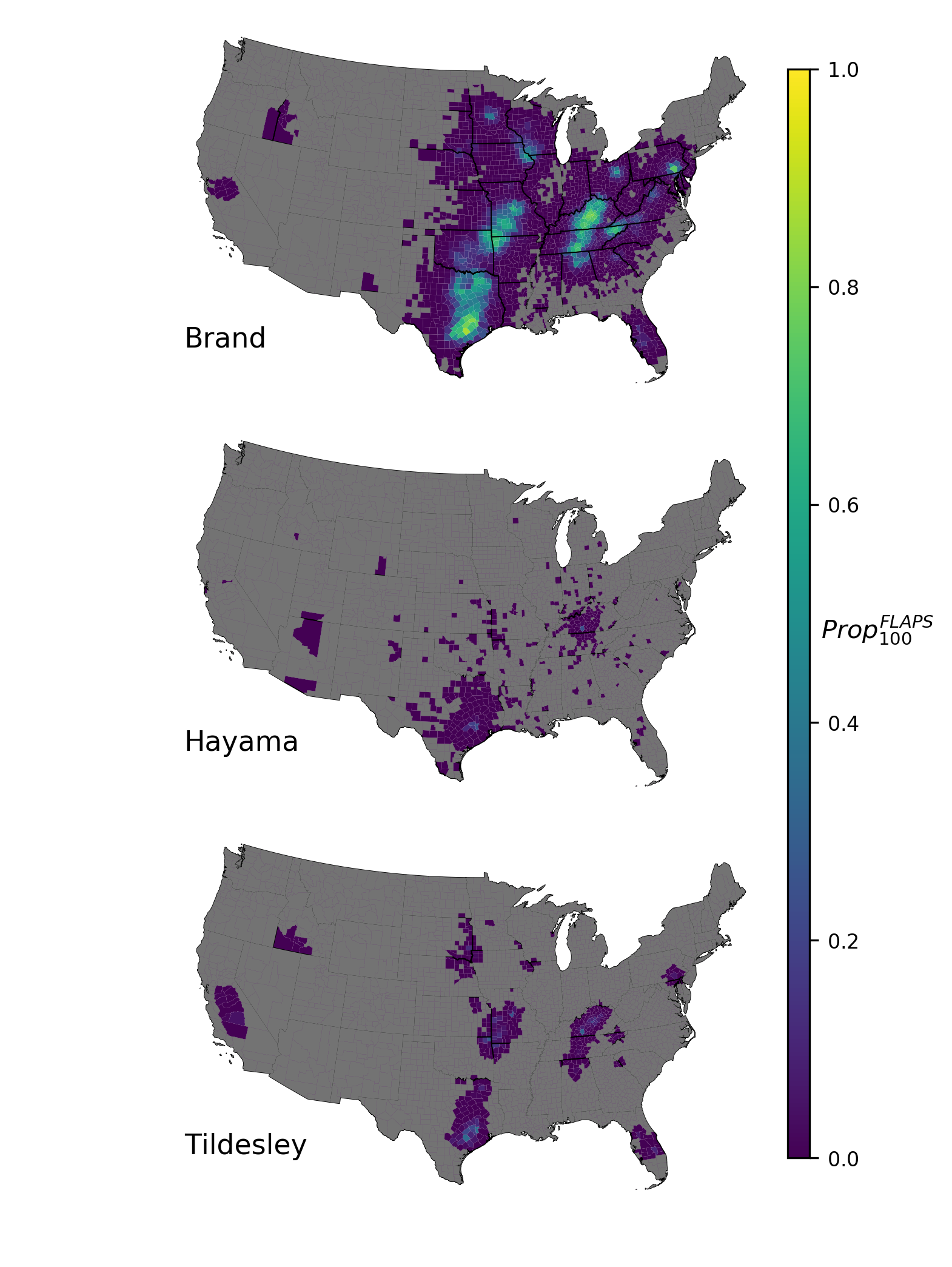

Supplement: S11 Fig — Transmissibility x1, FLAPS configuration. Color indicates the proportion of simulated outbreaks that reach 100 infected premises or more out of 10,000 replicates if outbreak starts in this county. Grey indicates that no outbreak started in the county reached 100 infected premises. (TIFF) [file pcbi.1007641.s011.tiff]
