# Supplementary material for: Realistic assumptions about spatial locations and clustering of premises matter for models of foot-and-mouth disease spread in the United States
Source: PLoS Comput Biol. 2020 Feb 20;16(2):e1007641. doi: 10.1371/journal.pcbi.1007641 (PMC7053778; doi:10.1371/journal.pcbi.1007641)
Supplement: S1 Table — Each combination of kernel, transmissibility scale factor and spatial premises distribution is based on 480,000 seeded outbreaks. (PDF) [file pcbi.1007641.s012.pdf]

		Proportion of outbreaks			
		Random		FLAPS	
	Kernel	Transm. x 1	Transm. x 5	Transm.x 1	Transm.x 5
Max 10,000 infected (county subset)	Tildesley	0.00001	0.08728	0.00002	0.14239
	Hayama	0.00001	0.0099	0.00003	0.0425
	Brand	0	0.45035	0.00003	0.49169
