# Supplementary material for: Realistic assumptions about spatial locations and clustering of premises matter for models of foot-and-mouth disease spread in the United States
Source: PLoS Comput Biol. 2020 Feb 20;16(2):e1007641. doi: 10.1371/journal.pcbi.1007641 (PMC7053778; doi:10.1371/journal.pcbi.1007641)
Supplement: S2 Table — Coefficient of discrimination (CoD), relative change in CoD (ΔCoD) and estimated coefficients of the predictors of logistic regression models predicting the probability of reaching 100 infected premises. The predictors are the county-level clustering at a spatial scale relevant for the kernel, Kwr^ (clustering), the type of configuration (random or FLAPS, landscape conf.), the logarithm of the average premises size in the seeded county (avg. prem. size), the logarithm of the number of premises in the seeded county (N. prem.) and the logarithm of the premises size of the seeded premises, (seeded size). (PDF) [file pcbi.1007641.s013.pdf]

	Removed variable	*CoD*	*ΔCoD*	*Intercept*	*Clustering*	*Landsc. conf.*	*Avg. prem. size*	*N. prem.*	*Seeded size*	*Real. 1*	*Real. 2*	*Real. 3*	*Real. 4*	*Real. 5*	*Real. 6*	*Real. 7*	*Real. 8*	*Real. 9*
Brand	*None (full model)*	0.373	0	-0.018	2.525	0.38	0.291	-0.17	0.368	-0.011	-0.023	-0.032	-0.018	-0.039	-0.038	-0.03	-0.028	-0.008
	Clustering	0.153	0.59	-0.014	-	0.315	-0.66	2.025	0.348	0.015	0.002	0.003	0.014	0.004	0.004	0.014	0	0.005
	Landsc. conf.	0.37	0.008	-0.018	2.516	-	0.292	-0.171	0.366	-0.012	-0.023	-0.033	-0.019	-0.042	-0.039	-0.032	-0.028	-0.008
	Avg. prem. size	0.37	0.008	-0.01	2.459	0.379	-	-0.116	0.413	-0.009	-0.022	-0.03	-0.016	-0.038	-0.035	-0.027	-0.027	-0.006
	N. prem	0.372	0.003	-0.017	2.439	0.38	0.254	-	0.37	-0.01	-0.022	-0.031	-0.017	-0.037	-0.036	-0.028	-0.027	-0.008
	Seeded size	0.365	0.022	-0.012	2.513	0.375	0.463	-0.186	-	-0.011	-0.023	-0.033	-0.019	-0.039	-0.039	-0.031	-0.029	-0.009
Hayama	*None (full model)*	0.117	0	-0.034	1.388	4.287	0.88	1.078	0.319	0.052	-0.014	0.059	0.046	0.129	-0.036	0.067	0.021	0.054
	Clustering	0.014	0.876	-0.054	-	7.441	-0.298	3.919	0.282	0.059	0.025	0.071	0.045	0.123	0.091	0.105	0.061	0.037
	Landsc. conf.	0.119	-0.02	-0.011	1.439	-	0.889	1.039	0.321	0.051	-0.016	0.06	0.047	0.13	-0.041	0.064	0.022	0.057
	Avg. prem. size	0.115	0.014	-0.034	1.36	4.325	-	0.755	0.368	0.064	-0.009	0.069	0.062	0.128	-0.023	0.088	0.041	0.068
	N. prem	0.113	0.033	-0.03	1.543	4.083	0.249	-	0.323	0.075	0.01	0.092	0.075	0.157	-0.039	0.078	0.061	0.099
	Seeded size	0.112	0.041	-0.03	1.379	4.299	1.009	1.09	-	0.055	-0.01	0.064	0.052	0.138	-0.035	0.071	0.027	0.061
Tildesley	*None (full model)*	0.128	0	-0.021	1.848	2.916	1.331	0.394	0.607	0.063	-0.026	0.004	0.157	0.024	0.025	0.022	-0.031	0.03
	Clustering	0.027	0.79	-0.031	-	4.457	0.196	2.638	0.539	0.053	0.009	0.039	0.163	0.067	0.046	0.048	0.015	0.067
	Landsc. conf.	0.129	-0.009	-0.015	2.065	-	1.434	0.24	0.612	0.077	-0.01	0.017	0.167	0.019	0.034	0.026	-0.017	0.046
	Avg. prem. size	0.112	0.131	-0.017	1.627	3.105	-	0.147	0.834	0.051	-0.036	0.002	0.149	0.028	0.017	0.018	-0.037	0.029
	N. prem	0.128	0.003	-0.02	1.947	2.824	1.252	-	0.606	0.069	-0.028	0.005	0.16	0.024	0.022	0.023	-0.032	0.026
	Seeded size	0.116	0.1	-0.015	1.822	2.918	1.566	0.372	-	0.061	-0.026	-0.001	0.156	0.018	0.024	0.019	-0.034	0.027
