# Supplementary material for: Realistic assumptions about spatial locations and clustering of premises matter for models of foot-and-mouth disease spread in the United States
Source: PLoS Comput Biol. 2020 Feb 20;16(2):e1007641. doi: 10.1371/journal.pcbi.1007641 (PMC7053778; doi:10.1371/journal.pcbi.1007641)
Supplement: S3 Table — Coefficient of discrimination (CoD), relative change in CoD (ΔCoD) and estimated coefficients of the predictors of logistic regression models predicting the probability of reaching 100 infected premises. The predictors are the county-level clustering at a spatial scale relevant for the kernel, Kwr^ (clustering), the type of configuration (random or FLAPS, landscape conf.), the logarithm of the average premises size in the seeded county (avg. prem. size), the logarithm of the number of premises in the seeded county (N. prem.) and the logarithm of the premises size of the seeded premises, (seeded size). (PDF) [file pcbi.1007641.s014.pdf]

	Removed variable	*CoD*	*ΔCoD*	*Intercept*	*Clustering*	*Landsc. conf.*	*Avg. prem. size*	*N. prem.*	*Seeded size*	*Real. 1*	*Real. 2*	*Real. 3*	*Real. 4*	*Real. 5*	*Real. 6*	*Real. 7*	*Real. 8*	*Real. 9*
Brand	*None (full model)*	0.533	0	0.023	3.179	0.173	0.327	-0.176	0.45	-0.02	-0.018	-0.022	-0.022	-0.034	-0.027	-0.033	-0.024	-0.016
	Clustering	0.274	0.486	0.01	-	0.2	-0.476	1.442	0.379	-0.001	0.004	0.003	-0.002	-0.005	0.007	0	-0.001	-0.002
	Landsc. conf.	0.532	0.001	0.023	3.178	-	0.328	-0.176	0.449	-0.02	-0.018	-0.022	-0.022	-0.034	-0.027	-0.033	-0.025	-0.016
	Avg. prem. size	0.526	0.012	0.012	2.944	0.178	-	-0.037	0.562	-0.015	-0.011	-0.015	-0.017	-0.031	-0.019	-0.027	-0.017	-0.011
	N. prem	0.532	0.002	0.021	3.035	0.173	0.283	-	0.453	-0.019	-0.016	-0.02	-0.02	-0.032	-0.025	-0.031	-0.023	-0.015
	Seeded size	0.519	0.026	0.015	3.126	0.166	0.505	-0.196	-	-0.022	-0.021	-0.024	-0.023	-0.033	-0.028	-0.034	-0.027	-0.018
Hayama	*None (full model)*	0.329	0	-0.011	1.453	0.061	-0.087	0.476	0.439	0.032	0.009	0.009	0.028	0.012	-0.018	0.02	0.005	0.02
	Clustering	0.174	0.47	-0.013	-	1.624	-0.399	1.806	0.383	0.04	0.022	0.024	0.037	0.022	-0.002	0.03	0.021	0.027
	Landsc. conf.	0.329	-0.001	-0.011	1.47	-	-0.083	0.461	0.439	0.032	0.009	0.009	0.028	0.012	-0.018	0.02	0.005	0.02
	Avg. prem. size	0.329	-0.001	-0.011	1.463	0.043	-	0.478	0.417	0.03	0.008	0.007	0.027	0.011	-0.019	0.019	0.003	0.018
	N. prem	0.327	0.006	-0.011	1.658	-0.207	-0.087	-	0.437	0.032	0.009	0.008	0.028	0.013	-0.021	0.022	0.003	0.019
	Seeded size	0.318	0.034	-0.01	1.437	0.047	0.115	0.464	-	0.029	0.007	0.006	0.026	0.011	-0.021	0.017	0.002	0.017
Tildesley	*None (full model)*	0.438	0	-0.016	2.248	-0.146	0.852	0.123	0.697	-0.027	-0.029	-0.029	-0.028	-0.033	-0.027	-0.031	-0.025	-0.022
	Clustering	0.263	0.399	-0.014	-	0.589	0.063	1.972	0.58	-0.006	-0.007	-0.006	-0.004	-0.001	-0.004	-0.003	-0.004	-0.008
	Landsc. conf.	0.437	0.001	-0.016	2.213	-	0.84	0.159	0.697	-0.026	-0.028	-0.029	-0.028	-0.032	-0.027	-0.031	-0.025	-0.022
	Avg. prem. size	0.405	0.075	-0.009	1.777	0.007	-	0.343	0.909	-0.019	-0.018	-0.02	-0.019	-0.024	-0.016	-0.022	-0.016	-0.014
	N. prem	0.438	0	-0.016	2.322	-0.174	0.863	-	0.697	-0.027	-0.029	-0.03	-0.029	-0.034	-0.028	-0.032	-0.026	-0.022
	Seeded size	0.4	0.086	-0.011	2.137	-0.152	1.082	0.103	-	-0.025	-0.027	-0.028	-0.026	-0.032	-0.026	-0.03	-0.024	-0.021
