# Supplementary material for: Realistic assumptions about spatial locations and clustering of premises matter for models of foot-and-mouth disease spread in the United States
Source: PLoS Comput Biol. 2020 Feb 20;16(2):e1007641. doi: 10.1371/journal.pcbi.1007641 (PMC7053778; doi:10.1371/journal.pcbi.1007641)
Supplement: S4 Table — Relative correlation coefficients from linear regression of independent variables vs dependent variable number of time steps to reach l infected premises where l is 10, or 100. The same set of independent variables were used as in the logistic regression. For each kernel, only the subset of simulations that reached L were used in the regression and only the results from the simulations based on transmissibility x5 were used. The number of time steps (days) required to reach 100 infected premises was highly affected by clustering. High clustering in the seeded county generally led to faster development of the outbreak, and a similar, although less substantial, relationship with landscape configuration is seen. We point out that one caveat to this analysis is that it does not take into account the higher frequency with which the simulations with FLAPS landscape configurations reached l. (PDF) [file pcbi.1007641.s015.pdf]

	Time steps to reach 10 infected			Time steps to reach 100 infected		
	Brand	*Hayama*	*Tildesley*	*Brand*	*Hayama*	*Tildesley*
Clustering	-4.45	-5.47	-6.26	-11.55	-14.03	-20.83
Landsc. conf. (is FLAPS)	-0.38	-1.21	-0.40	-0.94	-19.79	-1.79
N. prem.	-0.08	0.14	0.07	0.79	1.82	1.28
Seeded size	-0.99	-1.47	-2.52	-1.06	-1.68	-2.48
Avg. prem. size	-0.51	-0.43	-2.81	-0.79	2.92	-9.92
Realization 2	0.04	-0.02	0.09	0.11	0.21	0.32
Realization 3	0.05	-0.01	0.12	0.14	-0.02	0.48
Realization 4	0.04	-0.08	0.08	0.10	-0.20	0.25
Realization 5	0.06	-0.01	0.11	0.20	0.01	0.26
Realization 6	0.05	0.07	0.10	0.16	0.04	0.44
Realization 7	0.06	-0.07	0.12	0.17	0.03	0.41
Realization 8	0.04	-0.01	0.11	0.11	0.17	0.39
Realization 9	0.02	-0.03	0.07	0.07	0.21	0.24
Realization 10	0.03	-0.04	0.08	0.11	-0.06	0.22
