# Supplementary material for: Realistic assumptions about spatial locations and clustering of premises matter for models of foot-and-mouth disease spread in the United States
Source: PLoS Comput Biol. 2020 Feb 20;16(2):e1007641. doi: 10.1371/journal.pcbi.1007641 (PMC7053778; doi:10.1371/journal.pcbi.1007641)
Supplement: S1 Appendix — (PDF) [file pcbi.1007641.s018.pdf]

S1 Appendix – Maximal outbreak size.

In order to examine the effect of clustering on the potential outbreak size in the absence of any control we performed simulations on a subset of 48 counties, with no cutoff in number of infected premises (see main paper). Instead we let the simulations carry on until the outbreak died out on its own. From each of the 5,000 replicates of each combination of kernel, landscape configuration and seeded county we selected the replicates that reached at least 10 infected premises. For each of the resulting subsets the average number of infected premises was calculated. The histogram below shows how this average changes in relative terms when using the FLAPS landscapes compared to when using the randomized landscapes.

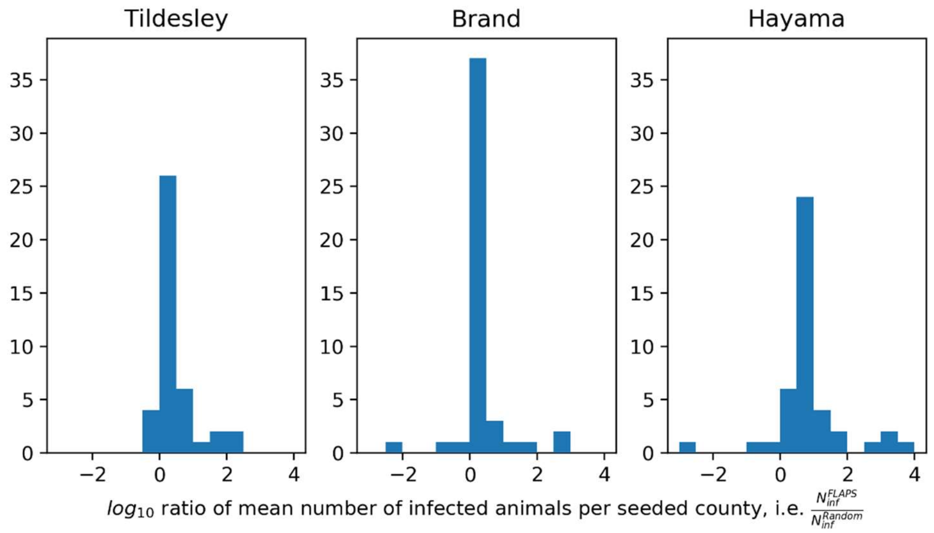

From the figure, it is clear that for most combinations of seeded county and kernel, the ratio is > 1.0, meaning that the outbreak size was on average larger when using the FLAPS configurations than when using the random configurations. This may not be surprising, but it illustrates that the clustering of the landscape will have an effect beyond just increasing the probability of an outbreak taking off. Or in other words, even when controlling for the fact that the outbreak takes off or not (it reaches at least 10 premises) the FLAPS configuration will increase outbreak sizes.

A few of the bars in the histogram clearly show that these is an increase in outbreak size with the random configuration (ratio < 1.0), something which may be surprising. These have reasonable explanations, though. For instance, the lowest value in the Hayama subplot is caused by purely stochastic reasons as for that specific case there were only two replicates out of 5,000 in each of the landscape configurations that reached above 10 infected premises, and one of the two for the random configuration became a very large outbreak with 49797 infected premises, while the others all just reached 13.

Another example is for the Brand kernel in which the county that was selected to seed in the state of Rhode Island consists of an island. When using FLAPS, the premises are concentrated to the land mass of that island. The randomization process used to create the random landscape configuration does not take into account water as long as it was inside the county borders, and so allowed premises to be placed in the water, bringing them closer to the premises situated on the mainland, increasing the risk that the outbreaks seeded here would take off. If anything this illustrates even more clearly the importance of using a sophisticated method such as FLAPS to simulate premises locations for outbreak simulations.
